# The Combination of a Donor–Acceptor TADF and a MR‐TADF Emitting Core Results in Outstanding Electroluminescence Performance

**DOI:** 10.1002/adma.202412761

**Published:** 2024-10-12

**Authors:** Dongyang Chen, Hui Wang, Dianming Sun, Sen Wu, Kai Wang, Xiao‐Hong Zhang, Eli Zysman‐Colman

**Affiliations:** ^1^ Institute of Functional Nano & Soft Materials (FUNSOM) Joint International Research Laboratory of Carbon‐Based Functional Materials and Devices Soochow University Suzhou Jiangsu 21523 P. R. China; ^2^ Organic Semiconductor Centre EaStCHEM School of Chemistry University of St Andrews St Andrews Fife KY16 9ST UK; ^3^ Jiangsu Key Laboratory for Carbon‐Based Functional Materials & Devices Soochow University Suzhou Jiangsu 215123 P. R. China; ^4^ Jiangsu Key Laboratory of Advanced Negative Carbon Technologies Soochow University Suzhou Jiangsu 21523 P. R. China

**Keywords:** efficiency roll‐off, Green emission, MR‐TADF, OLED, RISC

## Abstract

Here the utility and potential of an emitter design are demonstrated, consisting of a narrowband‐emitting multiresonant thermally activated delayed fluorescent (MR‐TADF) core that is decorated with a suitably higher energy donor‐acceptor TADF moiety. Not only does this D–A TADF group offer additional channels for triplet exciton harvesting and confers faster reverse intersystem crossing (RISC) kinetics but it also acts as a steric shield, insulating the emissive MR‐TADF core from aggregation‐caused quenching. Two emitters, **DtCzBN‐CNBT1** and **DtCzBN‐CNBT2**, demonstrate enhanced photophysical properties leading to outstanding performance of the organic light‐emitting diodes (OLEDs). **DtCzBN‐CNBT2**, containing a D–A TADF moiety, has a faster *k*_RISC_ (1.1 × 10^5^ s^−1^) and higher photoluminescence quantum yield (*Φ*_PL_: 97%) compared to **DtCzBN‐CNBT1** (0.2 × 10^5^ s^−1^, *Φ*_PL_: 90%), which contains a D–A moiety that itself is not TADF. The sensitizer‐free OLEDs with **DtCzBN‐CNBT2** achieve a record‐high maximum external quantum efficiency (EQE_max_) of 40.2% and showed milder efficiency roll‐off (EQE_1000_ of 20.7%) compared to the **DtCzBN‐CNBT1**‐based devices (EQE_max_ of 37.1% and EQE_1000_ of 11.9%).

## Graphical abstract

The ToC illustrates the molecular structure of **DtCzBN‐CNBT2** along with a brief overview of the performance of the corresponding OLED. The green and blue‐colored moieties represent the MR‐TADF emitting core and the pendant D–A TADF shield, respectively.

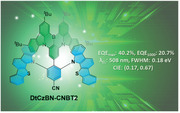

## 1 Introduction

Multiresonant thermally activated delayed fluorescence (MR‐TADF) compounds hold great potential as emitters in organic light‐emitting diodes (OLEDs) as devices with these materials can achieve simultaneously high efficiencies and saturated colors.^[^ ^1^ ^]^ Distinct from traditional donor‐acceptor (D–A) TADF emitters, MR‐TADF emitters typically are p‐ and n‐doped nanographenes.^[^ ^2^ ^]^ Their rigid molecular structures suppress vibronic modes and result in small geometric reorganization in the excited states, resulting in narrow full width at half maxima (FWHMs, typically <50 nm) and small Stokes shifts of less than 30 nm.^[^ ^3^ ^]^ The origin of the TADF in MR‐TADF emitters results from their distinct frontier molecular orbital (FMO) distributions, leading to an alternating pattern of increasing and decreasing electron density in the excited states compared to the ground state that is responsible for the suitably small singlet‐triplet energy gap, Δ*E*_ST_.^[^ ^1^, ^4^ ^]^ This distribution pattern leads to these materials emitting from a short‐range charge‐transfer (SRCT) excited state.^[^ ^1^, ^5^ ^]^ Moreover, as the electron density globally resides across the same atoms, the oscillator strength for the SRCT transitions is high, resulting in fast radiative decay rate constants (*k*_r_), manifested in high photoluminescence quantum yields (Φ_PL_).^[^ ^6^ ^]^ However, in most MR‐TADF systems, the reverse intersystem crossing (RISC) rate constant (*k*_RISC_) typically struggles to reach even 10^4^ s^−1^,^[^ ^7^ ^]^ which is much slower than those of most advanced D–A TADF emitters.^[^ ^8^ ^]^ Such slow *k*_RISC_ values are disadvantageous for efficient triplet harvesting, and the accumulation of long‐lived triplet excitons, especially at high current densities, are responsible for deleterious triplet exciton‐related annihilation processes, such as triplet‐polaron annihilation (TPA)^[^ ^9^ ^]^ and triplet‐triplet annihilation (TTA),^[^ ^10^ ^]^ that give rise to lower efficiencies and severe efficiency roll‐off in the device.^[^ ^11^ ^]^ Recently, there have been a few reports of MR‐TADF emitters that have fast *k*_RISC_ of over 10^5^ s^−1^, and their use in devices translated into OLEDs exhibiting high efficiency and mild efficiency roll‐off.^[^ ^12^ ^]^ However, these emitters typically suffer from aggregation‐caused quenching even at low concentrations, and emission broadening, both of which unduly affect the device performance.^[^ ^12a–d^ ^]^

**Figure 1:**
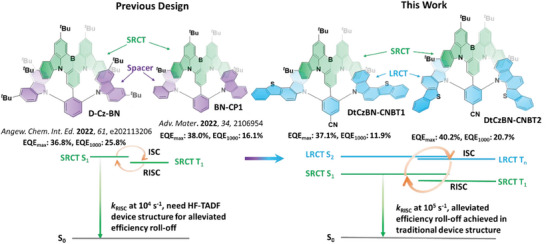
The molecular design strategy for **DtCzBN‐CNBT1** and **DtCzBN‐CNBT2**.

A more widely used solution to tackle the drop in performance in the device due to the use of an MR‐TADF emitter with too slow *k*_RISC_ is the addition of a fast *k*_RISC_ D–A TADF material within the emissive layer (EML) that acts as a sensitizer, i.e., so‐called hyperfluorescent OLEDs (HF‐OLEDs). In these devices, triplet excitons are first formed on the assistant dopant that then rapidly convert to singlets before being transferred to the MR‐TADF terminal emitter via Förster resonance energy transfer (FRET), thus leading to more efficient triplet harvesting in the device.^[^ ^13^ ^]^ Taking a benchmark MR‐TADF emitter **DtCzBN** (also known as **BCz‐BN**) as an example (**Figure** **1**), when the OLED was fabricated containing a conventional binary EML with 1 wt.% **DtCzBN** in 3,3′‐di(9*H*‐carbazol‐9‐yl)‐1,1′‐biphenyl (mCBP) host, although a high maximum external quantum efficiency (EQE_max_) of 21.6% was achieved, the efficiency dropped precipitously to 5.3% at a luminance of 1000 cd m^−2^.^[^ ^14^ ^]^ By contrast, the device possessing a ternary emissive layer containing 20 wt.% of the TADF sensitizer CTPCF3 not only showed a higher EQE_max_ of 27.5%, but more importantly, the efficiency roll‐off was much reduced, with the EQE_1000_ maintained at 24.1%.^[^ ^14^ ^]^ However, as the kinetics of FRET are largely dependent on the spectral overlap and the distance between the energy donor (the sensitizer) and acceptor (the terminal emitter), precise control of their concentration within the ternary EML is needed for optimal device performance. Here, we propose a molecular design where the functionalities of the assistant dopant and terminal emitter are embedded within a single molecule, thus ensuring efficient FRET between the two.

In this work, to realize such a molecular design, we connected a high‐energy D–A TADF moiety to the MR‐TADF‐emitting core **DtCzBN** in order to introduce multiple exciton‐harvesting channels akin to conventional HF systems. Two proof‐of‐concept emitters **DtCzBN‐CNBT1** and **DtCzBN‐CNBT2**, respectively, employ two bis(benzothienocarbazol‐5‐yl) benzonitrile isomers (**CNBT1** and **CNBT2** (Figure 1) as the D‐A‐type moieties, which are substituted onto the **DtCzBN** core and adopt a strongly twisted conformation. The **CNBT1** and **CNBT2** moieties not only sterically protect the emissive **DtCzBN** core thereby mitigating quenching at higher concentrations, a strategy that has been demonstrated to be effective in improving OLED performance at high emitter doping concentrations,^[^ ^15^ ^]^ and also contribute to a more efficient exciton harvesting by introducing additional RISC channels. In particular, **CNBT2** itself is TADF, with an associated Δ*E*_ST_ of 0.28 eV and a delayed lifetime (*τ*_d_) of 159 µs in 15 wt.% doped films in PhCzBCz, while **CNBT1** has a larger Δ*E*_ST_ of 0.36 eV and there is no observed delayed fluorescence in the doped PhCzBCz film. Both **DtCzBN‐CNBT1** and **DtCzBN‐CNBT2** show narrowband emission peaking at λ_PL_ of ca. 500 nm. Benefiting from the auxiliary D–A TADF moiety **CNBT2**, **DtCzBN‐CNBT2** has a five‐fold faster *k*_RISC_ of 1.1 × 10^5^ s^−1^ and higher *Φ*_PL_ of 97% than **DtCzBN‐CNBT1** (*k*_RISC_ of 2.0 × 10^4^ s^−1^, *Φ*_PL_ of 90%). The sensitizer‐free OLEDs with 15 wt.% **DtCzBN‐CNBT2** in PhCzBCz host showed green emission with CIE coordinates of (0.16, 0.67) and a record‐high EQE_max_ of 40.2%, while the EQE at 1000 cd m^−2^ was 20.7%. The **DtCzBN‐CN BT1**‐based device showed a comparably high EQE_max_ of 37.1% at CIE coordinates of (0.17, 0.66); however, the device exhibited severe efficiency roll‐off, with the EQE reducing to 11.9% at 1000 cd m^−2^ due to the slower *k*_RISC_ of the emitter. The state‐of‐the‐art device performance using **DtCzBN‐CNBT2** validates the effectiveness of the emitter design, which simultaneously boost *k*_RISC_, Φ_PL_, and maintains the narrowband emission associated with MR‐TADF materials.

## 2 Results and Discussion

### 2.1 Synthesis

The synthesis is outlined in Scheme S1 (Supporting Information). The intermediate **DtCzBN‐Bpin** was reacted with 4‐bromo‐3,5‐difluorobenzonitrile under Suzuki‐Miyaura cross‐coupling conditions to afford **DtCzBN‐CNF** in 70% yield. **DtCzBN‐CNF2** was then reacted with 5*H*‐benzo[4,5]thieno[3,2‐c]carbazole (**BT1**) or 12*H*‐benzo[4,5]thieno[2,3‐a]carbazole (**BT2**) under S_N_Ar conditions to obtain the target compounds **DtCzBN‐CNBT1** and **DtCzBN‐CNBT2** in 60 and 55%, respectively. The two emitters were purified by silica gel chromatography followed by temperature gradient vacuum sublimation. The identity of **DtCzBN‐CNBT1** and **DtCzBN‐CNBT2** were ascertained using a combination of ^1^H and ^13^C NMR spectroscopy and high‐resolution mass spectrometry (Figures S1–S6, Supporting Information). Their purity was verified by elemental analysis and high‐performance liquid chromatography (Figures S7–S10, Supporting Information). Thermal gravimetric analysis and melting point determination revealed that both **DtCzBN‐CNBT1** and **DtCzBN‐CNBT2** have high melting points (*M*_p_) of over 400 °C and excellent thermal stability, reflected in degrading temperatures, *T*_d_ (at 5% weight loss), of 449 and 443 °C, respectively (Figure S14, Supporting Information).

### 2.2 Theoretical Calculations

**Figure 2:**
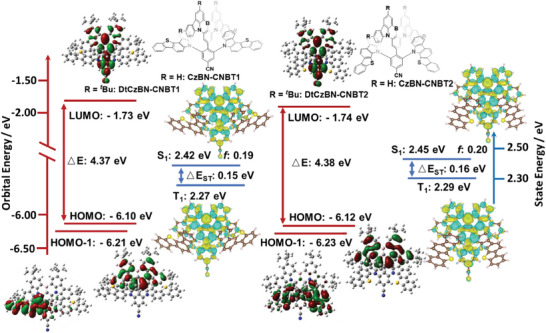
Calculated HOMO/LUMO distribution and energies at M062X/6‐31G(d,p) level of **DtCzBN‐CNBT1** and **DtCzBN‐CNBT2**, and S_1_ and T_1_ energies, and difference density plots at SCS‐(ADC)2/cc‐pVDZ level (blue: negative; green: positive), respectively of **CzBN‐CNBT1** and **CzBN‐CNBT2**.

Density functional theory (DFT) calculations were performed on **DtCzBN‐CNBT1** and **DtCzBN‐CNBT2** at the M062X/6‐31G(d,p) level to provide insight into the nature and energies of the low‐lying excited states and the frontier molecular orbitals. This level of theory was chosen as the M06X functional has a high ratio (54%) of Hartree–Fock‐like exchange, which has been shown to correlate with more accurate excited‐state energy predictions in D–A typed TADF emitters.^[^ ^16^ ^]^ As shown in **Figure** **2**, the HOMOs of **DtCzBN‐CNBT1** and **DtCzBN‐CNBT2** are located on the MR‐TADF **DtCzBN** core and have comparable energies of −6.10 and −6.12 eV, respectively, while the HOMO‐1 orbitals are stabilized by 0.11 eV and localized on one of the peripheral benzothienocarbazole donor moieties. The LUMOs of **DtCzBN‐CNBT1** and **DtCzBN‐CNBT2** are also located on the MR‐TADF core, and the electron density also extends to the pendant benzonitrile group. The orbital distribution pattern is complementary to that of the HOMO, residing largely on adjacent atoms within the **DtCzBN** core. Time‐dependent DFT calculations within the Tamm–Dancoff approximation (TDA‐DFT) were employed to interrogate both the nature and the energies of the excited states; additionally, spin‐orbit coupling matrix elements were determined between different combinations of low‐lying singlet/triplet excited states at the optimized T_1_ state geometry to assess the magnitude of the spin‐orbit coupling (SOC) between these states and thus the likely operational mechanism for RISC. Based on the HOMO‐LUMO transition that describes both the S_1_ and T_1_ states, both possess significant SRCT located on the MR‐TADF core, while the S_2_ states (3.48 eV for both emitters) are much better described as having long‐range charge transfer (LRCT) character transition from the pendant benzothienocarbazole to the benzonitrile acceptor. The S_1_ state (2.97 eV) of **DtCzBN‐CNBT2** is degenerate with the T_2_ state (2.95 eV), with $H_{\mathrm{SOC}}^{S1T2}$ of 0.45 cm^−1^ (Figure S18, Supporting Information), whereases for **DtCzBN‐CNBT1** the T_2_ energy (3.06 eV) is much higher than the S_1_ state (2.95 eV), while the magnitude of the $H_{\mathrm{SOC}}^{S1T2}$ of 0.41 cm^−1^ is similar (Figure S19, Supporting Information). The S_2_ states of **DtCzBN‐CNBT1** and **DtCzBN‐CNBT2** are also close in energy to those predicted for the S_1_/T_1_ states of, **CNBT1** (3.68/3.40 eV, Figure S13, Supporting Information) and **CNBT2** (3.66/3.41 eV, Figure S21, Supporting Information), which are the corresponding compounds containing only the pendant D–A moieties of **DtCzBN‐CNBT1** and **DtCzBN‐CNBT2**. This result indicates that the twisted structure can reduce the electronic communication between the MR‐TADF **DtCzBN** core and the pendant D–A moiety. For **DtCzBN‐CNBT1**, the S_2_ state (3.48 eV) lies close to three triplet states, T_5_ to T_7_ (3.38–3.45 eV, LRCT), with spin‐orbital coupling matrix element (SOCME) $H_{\mathrm{SOC}}^{S2T5}$ of 0.53, $H_{\mathrm{SOC}}^{S2T6}$ of 0.51, and $H_{\mathrm{SOC}}^{S2T7}$ of 0.49 cm^−1^, respectively. For **DtCzBN‐CNBT2**, the LRCT S_2_ state (3.43 eV) is degenerate with T_5_ (3.41 eV, SRCT) and T_6_ (3.46 eV, LRCT), with corresponding $H_{\mathrm{SOC}}^{S2T5}$ and $H_{\mathrm{SOC}}^{S2T6}$ of 1.97 and 1.00 cm^−1^, respectively. The larger $H_{\mathrm{SOC}}^{S2T5}$ and $H_{\mathrm{SOC}}^{S2T6}$ values for **DtCzBN‐CNBT2** are ascribed to the sulfur atom's participation in the S_2_ and T_6_ states (Figure S12, Supporting Information), which is not the case in **DtCzBN‐CNBT1** (Figure S11, Supporting Information), and this “heavy” atom effect from the sulfur atom in **DtCzBN‐CNBT2** boosts the SOC between the S_2_/T_5_ and S_2_/T_6_ states. The sufficiently higher‐lying LRCT S_2_ states of **DtCzBN‐CNBT1** and **DtCzBN‐CNBT2** guarantee that these states, even in polar media, will not be so stabilized as to affect the narrowband SRCT emission from the S_1_ states. Meanwhile, the small energy gap plus the large SOCME values between S_2_ and T_5_/T_6_ in **DtCzBN‐CNBT2** make S_2_ serve as a mediating channel to open a route for a higher‐lying RISC (hRISC) processes before internal conversion to the S_1_ state.

**Figure 3:**
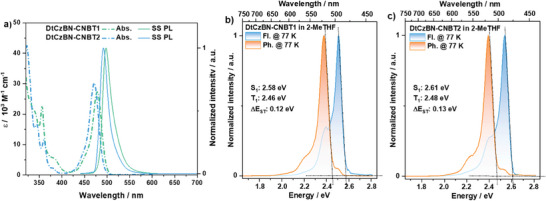
a) Absorption and steady‐state PL spectra (SS PL) of **DtCzBN‐CNBT1** and **DtCzBN‐CNBT2** in dilute toluene at room temperature (λ_exc_  =  360 nm); Steady‐state PL (298 and 77 K) and gated phosphorescence spectra (1–10 ms, 77 K) of b) **DtCzBN‐CNBT1** and c) **DtCzBN‐CNBT2** in 2‐MeTHF (λ_exc_  =  360 nm).

We also carried out SCS‐ADC2/cc‐PVDZ calculations on model emitters **CzBN‐CNBT1** and **CzBN‐CNBT2** (*tert*‐butyl groups omitted to reduce computational cost) based on the optimized ground‐state geometries at the M062X/6‐31G(d,p) level to obtain accurate predictions of the excited‐states energies, Δ*E*_ST_s, and electron densities,^[^ ^17^ ^]^ As shown in **Figure** **3**, both emitters have difference density patterns for both the S_1_ and T_1_ states that are reminiscent of SRCT states localized on the **DtCzBN** core and the benzonitrile substituent. The S_1_/T_1_ energies for **CzBN‐CNBT1** and **CzBN‐CNBT2** are 2.42/2.27 and 2.45/2.29 eV, respectively, resulting in near identical Δ*E*_ST_ values of 0.15 and 0.16 eV, respectively. The S_1_/T_1_ energies for **CzBN‐CNBT1** and **CzBN‐CNBT2** are moderately stabilized compared to those of **DtCzBN** (2.95/2.86 eV), which is ascribe to the presence of the *para*‐substituted electron‐accepting benzonitrile moiety.^[^ ^18^ ^]^

### 2.3 Electrochemistry

The energies of the HOMOs and LUMOs of **DtCzBN‐CNBT1** and **DtCzBN‐CNBT2** were inferred from cyclic voltammetry (CV) and differential pulse voltammetry (DPV) measurements in dimethylformamide (DMF) with 0.1 m tetrabutylammonium hexafluorophosphate, [*^n^* Bu_4_N]PF_6_, as the supporting electrolyte. Due to the presence of multiple electron‐accepting and electron‐donating moieties, the CV and DPV traces for both emitters show two irreversible reduction and oxidation waves, while for **DtCzBN** single irreversible reduction and oxidation waves are observed. The reduction and oxidation potentials, obtained from the peaks in the DPV, for **DtCzBN** are *E*_red_ = −1.77 and *E*_ox_ = 1.07 V, respectively, and the HOMO/LUMO energies are inferred as 5.42/2.58 eV (lit. reported HOMO of −5.40 eV via CV in DCM).^[^ ^14^ ^]^ For **DtCzBN‐CNBT1**, the two reduction potentials are *E*_red_ = −1.66 and −1.93 V, and the two oxidation potentials are *E*_ox_ = 1.10 and 1.21 V, respectively. For **DtCzBN‐CNBT2**, the reduction and oxidation waves have similar potentials to those of **DtCzBN‐CNBT1** at *E*_red_ = −1.66/−1.94 and *E*_ox_ = 1.11/1.21 V, respectively. The HOMO/LUMO energies for **DtCzBN‐CNBT1** and **DtCzBN‐CNBT2** are thus inferred to be −5.45/−2.69 and −5.46/−2.69 eV, respectively. The similar HOMO energies of **DtCzBN‐CNBT1**, **DtCzBN‐CNBT2,** and **DtCzBN**, match closely the DFT calculations as the HOMOs for **DtCzBN‐CNBT1** and **DtCzBN‐CNBT2** are mainly located on **DtCzBN** moieties. The moderately stabilized LUMO energies of **DtCzBN‐CNBT1** and **DtCzBN‐CNBT2** compared to **DtCzBN** are attributed to the presence of the *para*‐substituted electron‐withdrawing benzonitrile moiety, which leads to smaller HOMO/LUMO gaps of 2.76 and 2.77 eV, respectively, compared to 2.84 eV for **DtCzBN**.

### 2.4 Photophysics

Both **DtCzBN‐CNBT1** and **DtCzBN‐CNBT2** show intense low energy absorption bands in dilute toluene at room temperature associated with SRCT transitions, with molar extinction coefficients (ε) of 28 × 10^3^ (at 476 nm) and 32 × 10^3^ M^−1^ cm^−1^ (472 nm), respectively, both of which are close to the absorption of the emitting **DtCzBN** core (ε = 35 × 10^3^ M^−1^ cm^−1^ at 470 nm),^[^ ^3b^ ^]^ Figure 3a. The photoluminescence (PL) maxima (λ_PL_) of **DtCzBN‐CNBT1** and **DtCzBN‐CNBT2** in toluene are 498 and 492 nm, which are slightly red‐shifted compared to that of **DtCzBN** (λ_PL_ of 483 nm). Their emission is narrow, characterized by full width at half maxima, FWHM, of 22 and 21 nm, respectively; these are comparable to that of **DtCzBN** (FWHM = 23 nm). The narrowband emission and small Stokes shifts (both 20 nm) of both compounds indicate that the pendant donor‐acceptor group does not perturb the SRCT‐character of the emissive S_1_ state, even in medium‐to‐high polarity solvents such as THF, ethyl acetate, and acetonitrile (Figure S24, Supporting Information). The *Φ*_PL_ values of **DtCzBN‐CNBT1** and **DtCzBN‐CNBT2**in deaerated toluene are 83% and 85%, which are slightly lower compared to the *Φ*_PL_ of 98% of **DtCzBN**.^[^ ^3b^ ^]^ Meanwhile, the absorption spectra of **CNBT1** and **CNBT2** have weak low‐energy bands (ε of 2.4 × 10^3^ at 355 nm and 2.2 × 10^3^ M^−1^ cm^−1^ at 359 nm, respectively) in dilute toluene solution. Both **CNBT1** and **CNBT2** show deep blue emission, with λ_PL_ of 402 and 407 nm, that are weak, with *Φ*_PL_ of 12 and 9% in degassed toluene solution, respectively. The emission spectra of **CNBT1** and **CNBT2** showed moderate overlap with the absorption of **DtCzBN** (Figure S23, Supporting Information), with spectral overlap integrals^[^ ^19^ ^]^ estimated to be 1.2 × 10^13^ and 1.7 × 10^13^ M^−1^ cm^−1^ nm^4^, respectively. The energies of the S_1_/T_1_ states and Δ*E*_ST_ of **DtCzBN‐CNBT1** (2.58/2.46, 0.12 eV) and **DtCzBN‐CNBT2** (2.61/2.48, 0.13 eV) were determined from the corresponding onsets of the steady‐state fluorescence and phosphorescence spectra in 2‐methyl tetrahydrofuran (2‐MeTHF) glass at 77 K (Figure 3c), values that are energetically close to those of **DtCzBN** (2.63/2.50, 0.13 eV) in 2‐MeTHF glass (Figure S25, Supporting Information). The measured T_1_ state energies of **DtCzBN‐CNBT1** and **DtCzBN‐CNBT2** are likewise close to the TD‐DFT predicted values, while the S_1_ states are moderately stabilized compared to the predicted values. The similar measured energies indicate that the pedant **CNBT1** and **CNBT2** moieties do not significantly affect either the energies or nature of the lowest excited states of **DtCzBN‐CNBT1** and **DtCzBN‐CNBT2**, which are attributed to the higher energy (S_1_ >3.30, T_1_ >s2.89 eV) of the **CNBT1** and **CNBT2** moieties, as shown in Figure S25 (Supporting Information). **DtCzBN‐CNBT1**, **DtCzBN‐CNBT2**, **CNBT1**, and **CNBT2** exhibit prompt lifetimes, *τ*_p_, of 6.3, 4.5, 5.2, and 4.5 ns, respectively, in toluene solution and no delayed emission was observed (Figures S22, S27, Supporting Information).

**Figure 4:**
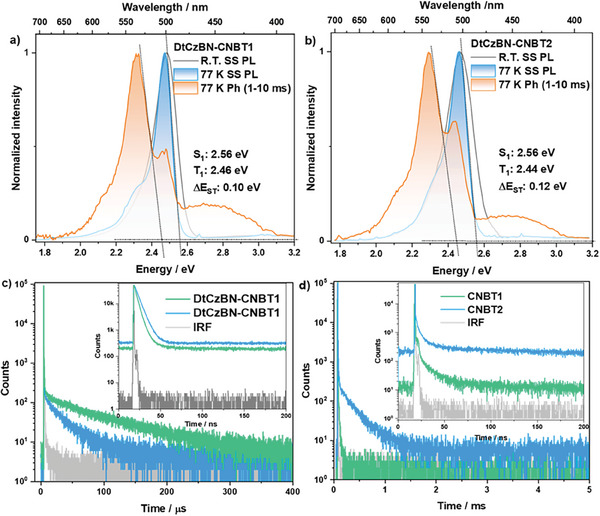
Steady‐state PL (298 and 77 K) and time‐gated phosphorescence spectra (2–10 ms, 77 K) of 15 wt.% doped films of a) **DtCzBN‐CNBT1** and b) **DtCzBN‐CNBT2** in PhCzBCz (λ_exc_  =  360 nm); Time‐resolved PL decays of 15 wt.% doped films in PhCzBCz of c) **DtCzBN‐CNBT1**/**DtCzBN‐CNBT2** and d) **CNBT1/CNBT2** within a 200 ns window (inset) and 5 ms window (λ_exc_ = 379 nm).

With a view to employing these compounds as emitters in OLEDs, the solid‐state photophysics of **DtCzBN‐CNBT1** and **DtCzBN‐CNBT2** were investigated in PhCzBCz as the host due to its suitably high triplet energy (E_T_ = 2.96 eV) and balanced carrier transport ability.^[^ ^20^ ^]^ Benefiting from the engineered encapsulated structure, the emission of **DtCzBN‐CNBT1** and **DtCzBN‐CNBT2** shows resistance to concentration quenching, as neither spectral broadening nor a decrease in *Φ*_PL_ was observed at concentrations up to 50 wt.% (Figure S29, Supporting Information). Across a doping concentration ranging from 1 to 50 wt.%, the λ_PL_ of **DtCzBN‐CNBT1** only slightly red‐shifts from 498 nm (1 wt.%) to 509 nm (50 wt.%), with a FWHM effectively remaining the same at 29 nm (0.14 eV) to 30 nm (0.15 eV), respectively. A similar behavior is observed for **DtCzBN‐CNBT2** where the λ_PL_ varies from 498 nm (1 wt.%) to 511 nm (50 wt.%) and the FWHM being 28 nm (0.13 eV) and 30 nm (0.15 eV), respectively. The modest red‐shifting of the PL was attributed to the compounds experiencing a more polar environment at higher doping concentrations. The highest *Φ*_PL_ values were achieved at 15 wt.% at 92% for **DtCzBN‐CNBT1** and 97% for **DtCzBN‐CNBT2** (97%); further, the *Φ*_PL_ values remain over 70% for **DtCzBN‐CNBT1** and **DtCzBN‐CNBT2** at a concentration as high as 50 wt.% (Figure S30, Supporting Information). The S_1_/T_1_ state energies and Δ*E*_ST_ values of the 15 wt.% doped films of **DtCzBN‐CNBT1** and **DtCzBN‐CNBT2** in PhCzBCz are 2.56/2.46/0.10 and 2.56/2.44/0.12 eV, respectively, determined from the difference in onsets of the steady‐state PL and phosphorescence spectra, which are close to the results measured in frozen 2‐MeTHF. The phosphorescence spectra of **DtCzBN‐CNBT1** and **DtCzBN‐CNBT2** have shoulder peaks at ≈510 nm, and a weak high‐energy, broad emission (400–470 nm) in doped PhCzBCz films (**Figure** **4**), which are ascribed to a combination of the residual delayed fluorescence from the MR‐TADF core and phosphorescence from the host.^[^ ^21^ ^]^ The time‐resolved PL decay traces of **DtCzBN‐CNBT1**, **DtCzBN‐CNBT2**, **CNBT1,** and **CNBT2** are shown in Figures 4c,d. Compounds **DtCzBN‐CNBT1** and **DtCzBN‐CNBT2** have prompt lifetimes, *τ*_p_, of 3.8 and 5.1 ns, and average delayed lifetimes, *τ*_d,avg_, of 75.5 and 15.3 µs, respectively. Considering that both compounds possess the same MR‐TADF moiety, the much shorter *τ*_d,avg_ of **DtCzBN‐CNBT2** must be due to the presence of the pendant donor‐acceptor moiety. Notably, the donor‐acceptor moieties **CNBT1** and **CNBT2** showed deep‐blue emission with λ_PL_ of 429 and 432 nm, and *Φ*_PL_ of 19 and 15%, respectively. The *τ*_p_ of **CNBT1** and **CNBT2** are similar at 2.7 and 3.5 ns, respectively, and only **CNBT2** exhibits a delayed emission, with *τ*_d,avg_ of 1.2 ms in 10 wt.% doped films in PhCzBCz. The origins of the TADF in **CNBT2** are due to the smaller Δ*E*_ST_ of 0.35 eV in the 15 wt.% doped film in PhCzBCz film (Figure S26, Supporting Information) and the larger predicted $H_{\mathrm{SOC}}^{S1T1}$ of 0.73 cm^−1^ benefiting from the sulfur atom's direct participation in the S_1_ and T_1_ states (Figure S21, Supporting Information). For **CNBT1**, TADF is not present due to a combination of the larger Δ*E*_ST_ of 0.41 eV and smaller $H_{\mathrm{SOC}}^{S1T1}$ of 0.24 cm^−1^ (Figure S21, Supporting Information) in this compound. The TADF character of **CNBT2** implies the presence of multiple RISC channels available in **DtCzBN‐CNBT2**, evidenced by the faster RISC rate constant (*k*_RISC_ = 1.1 × 10^5^ s^−1^) compared to the *k*_RISC_ of 2.0 × 10^4^ s^−1^ for **DtCzBN‐CNBT1** (**Table** **1**); a complete kinetics analysis is provided in the ESI. The FRET efficiency from **CNBT1**/**CNBT2** to **DtCzBN** was investigated in the systems of **DtCzBN**: **CNBT1** or **CNBT2**: PhCzBCz = 1:10:89. As shown in Figure S23 (Supporting Information) the emission at ≈430 nm from **CNBT1** or **CNBT2** disappears and the FRET efficiencies are calculated to be 96 and 98% for **CNBT1/DtCzBN** and **CNBT2/DtCzBN**, respectively.

**Table 1:** Key photophysical data of **DtCzBN‐CNBT1** and **DtCzBN‐CNBT2**.

Compound	λ_abs_ ^a)^/nm, ε^a)^/×10^3^ M^−1^ cm^−1^	λ_PL_ ^a)^, ^b)^/ nm	*Φ*_PL_ ^c)^/%	S_1_/T_1_/ Δ*E*_ST_ ^d)^/eV	*τ*_p_ ^b)^ / ns	*τ*_d_ ^b)^ / µs	*k*_r_ ^b)^/ 10^8^ s^−1^	*k*_RISC_ ^b)^/10^4^ s^−1^	*E*_HOMO_ ^e)^/ *E*_LUMO_ ^e)^/ *E*_g_ ^e)^/eV
**DtCzBN‐CNBT1**	476, 27	498, 502	60/90	2.56/2.46 /0.10	3.8	75.5	1.8	2.0	−5.45/‐2.69 /2.76
**DtCzBN‐CNBT2**	472, 30	492, 500	60/97	2.56/2.44 /0.12	5.1	15.3	1.6	10.6	−5.46/−2.69 /2.77

^a)^ Measured in diluted toluene (λ_exc_ = 340 nm);

^b)^ measured in 15 wt.% doped films in PhCzBCz (λ_exc_ = 379 nm);

^c)^ measured in 15 wt.% doped films in PhCzBCz using an integrating sphere under air/nitrogen (λ_exc _= 450 nm);

^d)^ S_1_ obtained from the onset of the SS PL spectrum at 77 K, T_1_ obtained from the onset of the time‐gated emission spectrum (1–10 ms) at 77 K (λ_exc_ = 340 nm);

^e)^ measured in DMF solution (E_HOMO_/_LUMO_ = −(E_ox_ / E_red_ vs Fc/Fc^+^ + 4.8) eV).

### 2.5 Electroluminescence Properties

**Figure 5:**
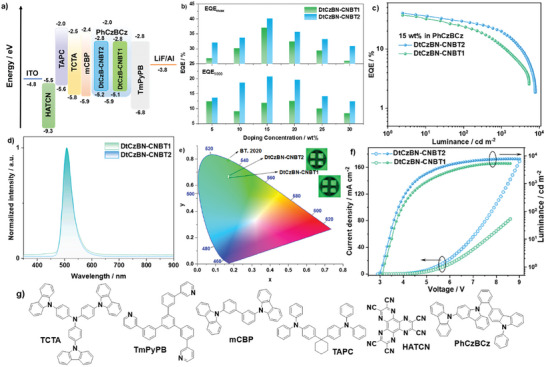
Optimized devices based on **DtCzBN‐CNBT1** and **DtCzBN‐CNBT2**: a) Energy‐level diagram for the OLEDs, b) EQE_max_/EQE_1000_ versus doping concentration characteristics, c) EQE‐luminance characteristics, d) EL spectra, e) devices emission images and their color coordinates in the CIE chromaticity diagram, f) Current density‐voltage‐luminance characteristics, g) chemical structures of the materials used in the devices.

**Figure 6:**
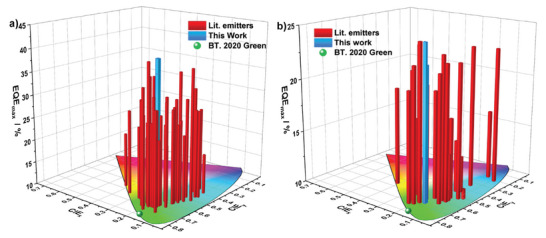
a) A comparison of CIE coordinates and EQE results for the best reported vacuum‐deposited green OLEDs based on MR‐TADF emitters; b) A comparison of EQE results for the solution‐processed OLEDs based on MR‐TADF emitters.

We next explored the potential of **DtCzBN‐CNBT1** and **DtCzBN‐CNBT2** as emitters in vacuum‐deposited OLEDs (VD‐OLEDs), fabricated using the following stack: ITO/HATCN (5 nm)/TAPC (30 nm)/TCTA (10 nm)/mCBP (10 nm)/emitter:PhCzBCz X wt.% (20 nm)/TmPyPB (40 nm)/LiF (1 nm)/Al (100 nm). For comparison, we also fabricated solution‐processed OLEDs (SP‐OLEDs) using the device architecture of ITO/PEDOT:PSS (35 nm)/emitter: PhCzBCz X wt.% (25 nm)/TmPyPB (40 nm)/LiF (1 nm)/Al (100 nm), (**Table** **2**; Figure S24, Supporting Information). For the VD‐OLEDs, HATCN acts as the hole injection layer, TAPC, and TCTA are used as hole transporting layers, *m*CBP is the exciton blocking layer, and TmPyPB is the electron‐transport material. PhCzBCz was selected as the host material, and to obtain optimal device performance, the doping concentrations of **DtCzBN‐CNBT1** and **DtCzBN‐CNBT2** were investigated from 5 to 30 wt.%. The device results are summarized in Table 2. Similar to their PL behavior, the electroluminescent maxima, λ_EL_, of the devices with **DtCzBN‐CNBT1** and **DtCzBN‐CNBT2** do not significantly change as the doping concentration increases from 5 to 30 wt.%, slightly bathochromically shifting from 504 to 510 nm, while the FWHM stays ≈at 38 nm. The maximum EQE (EQE_max_) for the device with **DtCzBN‐CNBT2** remained over 30.0% across all investigated doping concentrations, and the highest EQE_max_ of 40.2% was achieved at a concentration of 15 wt.% (**Figure** **5**b; Figure S31b, Supporting Information) with CIE coordinates of (0.17, 0.67). Although there are emitters with CIE_y_ of over 0.70, it is challenging to produce devices that simultaneously achieve high EQE_max_ (over 40%) and pure green emission that matches the BT 2020. standard [CIE of (0.17, 0.80), Figures 5e, **6**a]. For the device with **DtCzBN‐CNBT1,** the highest EQE_max_ of 37.1% (Figure 5b; Figure S31a, Supporting Information) was also achieved at this same doping concentration, with CIE coordinates of (0.17, 0.66). Moreover, thanks to its faster *k*_RISC_, the devices with **DtCzBN‐CNBT2** showed a decreased efficiency roll‐off of 48% at a brightness of 1000 cd m^−2^ at doping concentrations varying from 10 to 25 wt.%, while the devices with **DtCzBN‐CNBT1** all showed more severe efficiency roll‐off of 68% at 1000 cd m^−2^. The high EQE_max_ values for the devices with **DtCzBN‐CNBT1** and **DtCzBN‐CNBT2** originate from a combination of their near unity *Φ*_PL_ (90 and 97%) at the optimal concentration and the improved out‐coupling efficiencies resulting from their strongly horizontally oriented transition dipole moment (TDM), (measured in 15 wt.% doped PhCzBCz film to match the optimal concentration for *Φ*_PL_ and device result). As shown in Figure S34 (Supporting Information), the TDM of **DtCzBN‐CNBT1** and **DtCzBN‐CNBT2** are strongly horizontally orientated, with anisotropy factor, α, of 0.11 and 0.09, respectively. Thus, the theoretical EQE_max_ of the devices are 41.0 and 39.9%, comparable to the experimental EQE_max_ of 40.2 and 37.1%, respectively, assuming that in the devices the charge balance (*γ)* and the efficiency of producing radiative excitons (*η*_r_) both are unity. The high EQE_max_ and moderate efficiency roll‐off, plus the CIE_y_ of 0.67, make the OLEDs with **DtCzBN‐CNBT2** one of the best‐performing green‐emissive MR‐TADF devices (Figure 6a) to date; gratifyingly, there is no need to employ a bespoke TADF host or TADF assistant (Table S1, Supporting Information), which would be more desirable for industry application. These results also demonstrate the attractive value of our emitter design, which decorates a high‐energy TADF moiety onto the MR‐TADF emitter to simultaneously mitigate aggregation‐caused quenching and red‐shifting of the emission, and to provide multiple RISC channels that reduce efficiency roll‐off at high brightness.

**Table 2:** Device performance data of **DtCzBN‐CNBT1** and **DtCzBN‐CNBT2**.

Device	V_on_ /V	λ_EL_ /nm	CIE	FWHM /nm [eV]	EQE_max_/EQE_100_/ EQE_1000_/%	Lum_max_ /cd m^−2^
**DtCzBN‐CNBT1** 15 wt.% VD‐OLEDs	3.1	508	(0.17, 0.66)	38 (0.18)	37.1/23.4/11.9	5400
**DtCzBN‐CNBT1** 15 wt.% SP‐OLEDs	3.5	508	(0.17, 0.66)	39 (0.19)	22.4/12.9/4.8	3000
**DtCzBN‐CNBT2** 15 wt.% VD‐OLEDs	3.0	508	(0.17, 0.67)	37 (0.18)	40.2/29.5/20.7	7800
**DtCzBN‐CNBT2** 15 wt.% SP‐OLEDs	3.4	507	(0.16, 0.67)	38 (0.18)	24.4/14.9/5.6	4000

## 3 Conclusion

Two emitters (**DtCzBN‐CNBT1** and **DtCzBN‐CNBT2**) possessing, respectively, non‐TADF or TADF D–A moieties connected to the same MR‐TADF emitting core **DtCzBN** were rationally designed, modeled, synthesized, and characterized. The objective of the design strategy aimed to increase *k*_RISC_, improve triplet exciton harvesting, and maintain the high Φ_PL_, while simplifying the device fabrication for MR‐TADF emitters by removing the need for an external TADF sensitizer in order to achieve high‐performance devices. Thanks to the presence of the D–A TADF moiety, **DtCzBN‐CNBT2** demonstrated a shorter *τ*_d_ of 15.3 µs, a faster *k*_RISC_ of 1.1 × 10^5^ s^−1^, and a higher *Φ*_PL_ of 97% in a 15 wt.% doped film in PhCzBCz than **DtCzBN‐CNBT1**, which although having a similarly high *Φ*_PL_ of 92%, has a much longer *τ*_d_ of 75.5 µs and slower *k*_RISC_ of 2.0 × 10^4^ s^−1^. Sensitizer‐free OLEDs containing 15 wt.% **DtCzBN‐CNBT2** in PhCzBCz emitted green light with CIE coordinates of (0.16, 0.67) and achieved a record‐high average EQE_max_ of 40.2%, with an EQE of 20.7% at 1000 cd m^−2^. The device with **DtCzBN‐BT1CN** showed a similarly high average EQE_max_ of 37.1% and emitted with comparable CIE coordinates of (0.17, 0.66); however, the device experienced a significant efficiency roll‐off, with the EQE at 1000 cd m^−2^ dropping to 11.9% due to the slower *k*_RISC_ of the emitter. Solution‐processed OLEDs with **DtCzBN‐CNBT2** emitting at the same color point as the vacuum‐deposited analogs, showed lower efficiencies and stronger efficiency roll‐off, with an EQE_max_ of 24.4% and EQE_1000_ of 5.6%, while devices with **DtCzBN‐CNBT1** performed slightly worse (EQE_max_ of 22.3% and EQE_1000_ of 4.8%). The impressive vacuum‐deposited device performance using **DtCzBN‐CNBT2** validates the emitter design of integrating high‐energy TADF moieties as peripheral moieties decorating MR‐TADF emitting cores to enhance *k*_RISC_ without compromising the attractive photophysical properties endemic to MR‐TADF materials.

## Conflict of Interest

The authors declare no conflict of interest.

## Supporting information



Supporting Information

## Data Availability

The research data supporting this publication can be accessed at https://doi.org/10.17630/37b66d45‐2bd4‐468b‐98f6‐e4b4a542ce4e.
