# Supplementary material for: The Combination of a Donor–Acceptor TADF and a MR‐TADF Emitting Core Results in Outstanding Electroluminescence Performance
Source: Adv Mater. 2024 Oct 12;36(50):2412761. doi: 10.1002/adma.202412761 (PMC11635906; doi:10.1002/adma.202412761)
Supplement: Supplementary file 1 — Supporting Information [file ADMA-36-2412761-s001.pdf]

ADVANCED MATERIALS

Supporting Information

for *Adv. Mater.*, DOI 10.1002/adma.202412761

The Combination of a Donor–Acceptor TADF and a MR-TADF Emitting Core Results in Outstanding Electroluminescence Performance

Dongyang Chen, Hui Wang, Dianming Sun, Sen Wu, Kai Wang*, Xiao-Hong Zhang* and Eli Zysman-Colman**

The combination of a donor-acceptor TADF and a MR-TADF emitting core results in outstanding electroluminescence performance

Dongyang Chen,[a,b†] Hui Wang,[a†] Dianming Sun,[b] Sen Wu,[b] Kai Wang,*[a,c] Xiao-Hong Zhang,*[a,d]* and *Eli Zysman-Colman*[b]*

[a] Institute of Functional Nano & Soft Materials (FUNSOM), Joint International Research Laboratory of Carbon-Based Functional Materials and Devices, Soochow University, Suzhou, 21523, Jiangsu, PR China

[b] Organic Semiconductor Centre, EaStCHEM School of Chemistry, University of St Andrews, St Andrews, Fife, UK, KY16 9ST, U.K.

[c] Jiangsu Key Laboratory for Carbon-Based Functional Materials & Devices, Soochow University, Suzhou, 215123, Jiangsu, P. R. China

[d] Jiangsu Key Laboratory of Advanced Negative Carbon Technologies, Soochow University, Suzhou, 21523, Jiangsu, PR China

Contents

General methods **S3**

Experimental section **S7**

Computations **S23**

Photophysical characterization **S27**

Devices **S31**

References **S38**

General methods

General Synthetic Procedures.

The reagents and solvents were obtained from commercial sources and used as received unless otherwise stated. **DtCzBN-Bpin** was synthesized according to a literature procedure.[1] Air-sensitive reactions were done under a nitrogen atmosphere using Schlenk techniques. Dry solvents used in the reaction were obtained from a MBRAUN SPS5 solvent purification system. Flash column chromatography was carried out using silica gel (Silia-P from Silicycle, 60 Å, 40-63 μm). Analytical thin-layer-chromatography (TLC) was performed with silica plates with aluminium backings (250 μm with F-254 indicator). TLC visualization was accomplished by 254/365 nm UV lamp. HPLC was conducted on a Shimadzu LC-40 HPLC system. HPLC traces were performed using a Shim-pack GIST 3μm C18 reverse phase analytical column. ^{1}H and ^{13}C and NMR spectra were recorded on a Bruker Advance spectrometer (400 MHz for ^{1}H and 125 MHz for ^{13}C). The following abbreviations have been used for multiplicity assignments: “s” for singlet, “d” for doublet, “t” for triplet, “m” for multiplet, “dd” for doublet of doublets, “dt” for doublet of triplets. ^{1}H and ^{13}C NMR spectra were referenced to the solvent peaks). Melting points were measured using open-ended capillaries on an Electrothermal 1101D Mel-Temp apparatus and are uncorrected. High-resolution mass spectrometry (HRMS) was performed at Soochow University Mass Spectrometry Facility. Elemental analyses were performed by Dr. Joe Casillo at the University of Edinburgh.

Quantum chemical calculations

For the DFT calculations, the ground-state and excited singlet state were optimized using the M062X functional[2] and the 6-31G(d,p) basis set, followed by frequency calculations at the same level of theory to ensure that an energy minimum were reached. Excited-state calculations and optimizations were performed employing the Tamm-Dancoff approximation (TDA)[3] to Time-Dependent DFT (TD-DFT) using the same functional and basis set for ground states and excited states geometry optimization. The spin-orbital coupling matrix element (SOCME) values between excited states were obtained by PySOC calculation based on optimized triplet states’ geometry.[4] Gaussian 16[5] software was employed for the

calculations,[6] and GaussView 6.1 was used for visualization.[7] Vertical excited states were also calculated at the SCS-ADC2/cc-pVDZ level based on the PBE0/6-31G(d,p) ground-state optimized structure.[8] All calculations were submitted and processed using in-house developed software, Digichem ver 3,[9] which incorporates a number of publicly available software libraries, including: cclib[10] for parsing of result files, VMD[11]/Tachyon[12] for 3D rendering, Matplotlib[13] for drawing of graphs and Open Babel[14]/Pybel[15] for file interconversion, and the excited-state calculations were performed using Time-Dependent DFT with the same functional and basis set as for the ground-state geometry optimization in the gas phase.

Electrochemistry measurements

Cyclic Voltammetry (CV) analysis was performed on an Electrochemical Analyzer potentiostat model 620E from CH Instruments at a sweep rate of 100 mV/s. Differential pulse voltammetry (DPV) was conducted with an increment potential of 0.004 V and a pulse amplitude, width, and period of 50 mV, 0.05, and 0.5 s, respectively. Samples were prepared in DMF solutions, which were degassed by sparging with DMF-saturated nitrogen gas for 5 minutes prior to measurements. All measurements were performed using 0.1 M DMF solution of tetra-*n*-butylammonium hexafluorophosphate, $[^{n}Bu_4N]PF_6$. An Ag/Ag^+ electrode was used as the reference electrode while a platinum electrode and a platinum wire were used as the working electrode and counter electrode, respectively. The redox potentials are reported relative to a saturated calomel electrode (SCE) with a ferrocenium/ferrocene (Fc/Fc^+) redox couple as the internal standard (0.45 V vs SCE).[16] The HOMO and LUMO energies were determined using the relation $E_{HOMO}/_{LUMO} = -(E_{ox} / E_{red} + 4.8)$ eV, where E_{ox} and E_{red} are the onset of anodic and cathodic peak potentials, respectively calculated from DPV relative to Fc/Fc^+.[16]

Photophysical measurements

Optically dilute solutions of concentrations in the order of 10^{-5} or 10^{-6} M were prepared in HPLC grade solvent for absorption and emission analysis. Absorption spectra were recorded at room temperature on a Shimadzu UV-2600 double beam spectrophotometer. Molar absorptivity determination was verified by a linear regression analysis of values obtained from five independent solutions at varying

concentrations with absorbance ranging from 0.110 to 0.600 for **DtCzBN-CNBT1** and from 0.150 to 0.850 for **DtCzBN-CNBT2**. Aerated solutions were bubbled with compressed air for 5 minutes whereas degassed solutions were prepared via three freeze-pump-thaw cycles prior to emission analysis using an in-house adapted fluorescence cuvette, itself purchased from Starna. Steady-state emission and excitation spectra and time-resolved emission spectra were recorded at 298 K using an Edinburgh Instruments FS5 fluorimeter. Samples were excited at 340 nm for steady-state measurements and at 378 nm for time-resolved measurements Photoluminescence quantum yields for solutions were determined using the optically dilute method[17]in which four sample solutions with absorbances of ca. 0.10, 0.075, 0.050 and 0.025 at 360 nm were used. The Beer-Lambert law was found to remain linear at the concentrations of the solutions. For each sample, linearity between absorption and emission intensity was verified through linear regression analysis with the Pearson regression factor (R^2) for the linear fit of the data set surpassing 0.9. Individual relative quantum yield values were calculated for each solution and the values reported represent the slope obtained from the linear fit of these results. The quantum yield of the sample, Φ_{PL}, can be determined by the equation $\Phi_{PL} = (\Phi_r * \frac{A_r}{A_s} * \frac{I_s}{I_r} * \frac{n_s^2}{n_r^2})$,[17] where A stands for the absorbance at the excitation wavelength (λ_{exc}: 360 nm), I is the integrated area under the corrected emission curve and n is the refractive index of the solvent with the subscripts "s" and "r" representing sample and reference respectively. Φ_r is the absolute quantum yield of the external reference quinine sulfate (Φ_r = 54.6% in 1 *N* H_2SO_4).[18] The experimental uncertainty in the emission quantum yields is conservatively estimated to be 10%, though we have found that statistically we can reproduce Φ_{PL}s to 3% relative error.

An integrating sphere (Edinburgh Instruments FS5, SC30 module) was employed for the photoluminescence quantum yield measurements of thin film samples. The Φ_{PL} of the films were then measured in air and in N_2 by purging the integrating sphere with N_2 gas flow for 2 min. The photophysical properties of the film samples were measured using an Edinburgh Instruments FS5 fluorimeter. Time-resolved PL measurements of the thin films were carried out using the multi-channel scaling (MCS) and time-correlated single-photon counting (TCSPC) technique. The samples were excited at 379 nm by a pulsed laser and were kept in a vacuum of $< 8 \times 10^{-4}$ mbar. The singlet and

triplet state energies in 2-MeTHF glass and in doped film were determined from the onset values of the steady-state photoluminescence PL (SSPL) and phosphorescence spectra at 77 K. The singlet-triplet energy gap (ΔE_{ST}) was estimated from the difference in energy of the steady-state PL and phosphorescence spectra. The samples were excited by a xenon flashlamp emitting at 340 nm (EI FS5, SC-70). Phosphorescence spectra were measured with a time-gated window of 1-10 ms.

Fitting of the time-resolved luminescence measurements

Time-resolved PL measurements were fitted to a sum of exponentials decay model, with chi-squared (χ^2) values between 1 and 2, using the EI FS5 Each component of the decay is assigned with a weight, (w_i), which is the contribution of the emission from each component to the total emission.

The average lifetime was then calculated using the following expressions:

1. Two exponential decay model:

$$\tau_{AVG} = \tau_1 w_1 + \tau_2 w_2 \qquad \text{(S1)}$$

with weights defined as $w_1 = \frac{A1\tau_1}{A1\tau_1 + A2\tau_2}$ and $w_2 = \frac{A2\tau_2}{A1\tau_1 + A2\tau_2}$ where A_1 and A_2 are the preexponential-factors of each component.

2. Three exponential decay model:

$$\tau_{AVG} = \tau_1 w_1 + \tau_2 w_2 + \tau_3 w_3 \qquad \text{(S2)}$$

with weights defined as $w_1 = \frac{A1\tau_1}{A1\tau_1 + A2\tau_2 + A3\tau_3}$, $w_2 = \frac{A2\tau_2}{A1\tau_1 + A2\tau_2 + A3\tau_3}$ and $w_3 = \frac{A3\tau_3}{A1\tau_1 + A2\tau_2 + A3\tau_3}$ where A_1, A_2 and A_3 are the preexponential-factors of each component.

OLED Fabrication and Characterization

Device fabrication and measurement of EL characteristics. OLEDs were fabricated on the indium-tin oxide (ITO) coated transparent glass substrates with multiple layers. The ITO glass substrates have a thickness of ca. 100 nm and a sheet resistance of ca. 30 Ω per square and were cleaned with optical detergent, deionized water, acetone, and isopropanol successively and then dried in an oven. For

vacuum-evaporation OLEDs, the ITO substrates were exposed to UV ozone for 15 minutes at first. All the organic materials were thermal evaporated at a rate of 1 Å s^{-1} under a vacuum of ca. 10^{-5} Torr. Finally, LiF and Al were successively deposited at a rate of 0.1 Å s^{-1} and 5 Å s^{-1}, respectively. Four identical OLED devices were formed on each of the substrates and the emission area of 0.09 cm^2 for each device. For solution-process OLEDs, the ITO substrates were exposed to UV ozone for 15 minutes at first. Then, the PEDOT:PSS layer was prepared by spin-coating on the prepared ITO glass substrates and annealed at 120 °C for 20 min in air condition. Then, these glass substrates were transferred into nitrogen-filled glovebox to prepare the emissive materials layers. The host and guest were dissolved in chlorobenzene solvent (10 mg/mL) and the mixture were stirred at 50 °C before the spin-coating. The rest layers were then sequentially fabricated via vacuum-evaporation. The EL performances of the devices were measured with a PHOTO RESEARCH Spectra Scan PR 655 PHOTOMETER and a KEITHLEY 2400 Source Meter constant current source at room temperature.

Synthesis

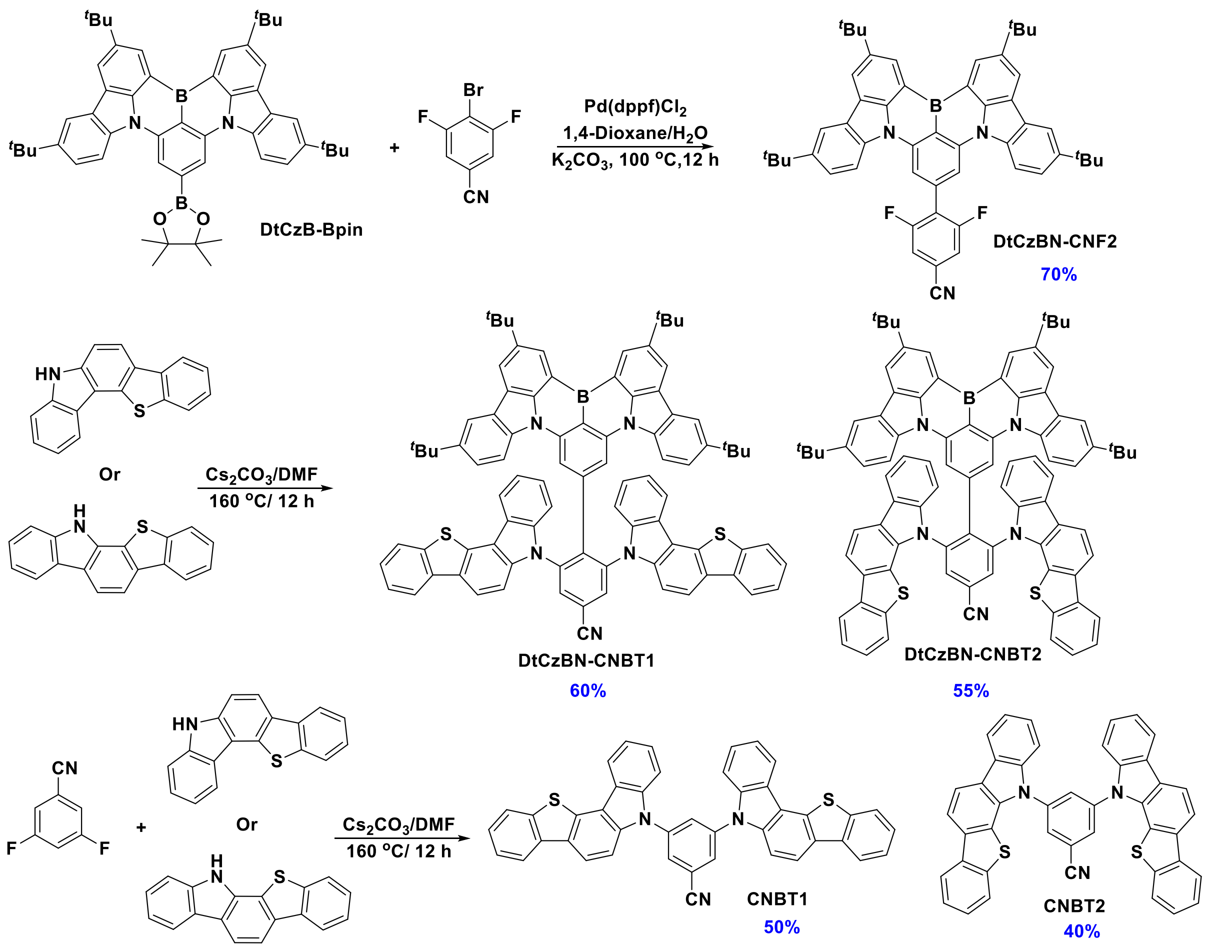

Scheme **S1**. Synthesis route of **DtCzBN-CNBT1**, **DtCzBN-CNBT2**, **CNBT1**, and **CNBT2**.

Synthesis of **DtCzBN-CNF2**

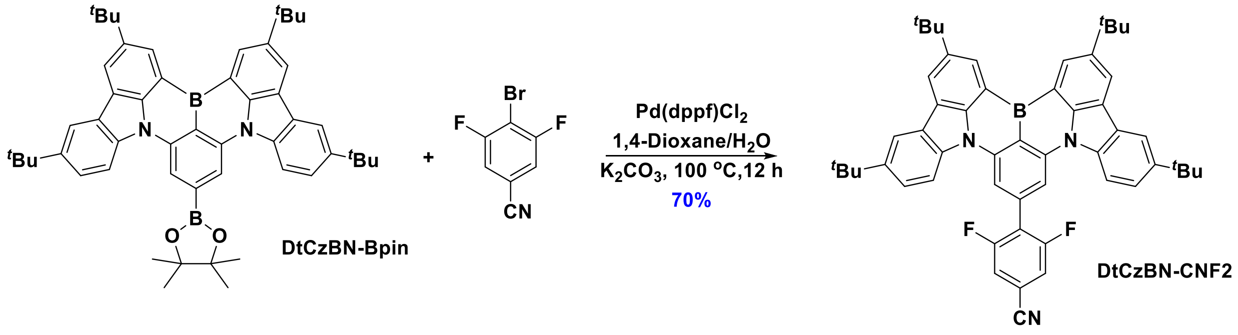

To a 50 mL two-neck flask, were added **DtCzBN-Bpin** (400 mg, 0.52 mmol 1 equiv.) were added 4-bromo-3,5-difluorobenzonitrile (171 mg, 0.78 mmol, 1.5 equiv.), and Pd(dppf)Cl_2 (30 mg, 0.05 mmol, 0.1 equiv.). The flask was degassed by three cycles of vacuum-nitrogen purging and Na_2CO_3 solution (2 M, 1 mL, 4 equiv.) and 1,4-dioxane (10 mL) were injected. The solution was bubbled by N_2 flow for

30 mins then heated at reflux for 12 h. The reaction was cooled to room temperature and diluted with DCM (100 mL). The organic layer was washed with water (3 × 50 mL) and then dried with anhydrous sodium sulfate. The solvents were removed under reduced pressure. The crude product was purified by column chromatography on silica gel (DCM:hexane = 1:3). The corresponding fractions were combined and concentrated under reduced pressure to afford bright orange solid. **Yield:** 70% (280 mg) **R_f:** 0.50 (DCM:hexane = 1:2) **Mp**; 389-392 °C. **^{1}H NMR (500 MHz, $CDCl_3$) δ (ppm):** 9.14 (s, 2H), 8.46 (d, *J* = 48.5 Hz, 4H), 8.30 (d, *J* = 9.7 Hz, 4H), 7.68 (d, *J* = 8.7 Hz, 2H), 7.50 (d, *J* = 6.4 Hz, 2H), 1.71 (s, 18H), 1.56 (s, 18H). **^{13}C NMR (126 MHz, CDCl3) δ (ppm):** 161.23, 159.28, 145.69, 145.02, 144.06, 141.63, 138.15, 129.88, 127.14, 124.61, 123.80, 121.59, 121.07, 117.43, 116.55, 116.31, 113.83, 112.98, 109.40, 35.23, 34.85, 32.19, 31.83. **HRMS** (MALDI-TOF) **(m/z)**: $[M+H]^+$ Calculated for **$C_{53}H_{50}B_1F_2N_3$:** 778.4139; **Found:** 778.4135.

Synthesis of **DtCzBN-CNBT1**

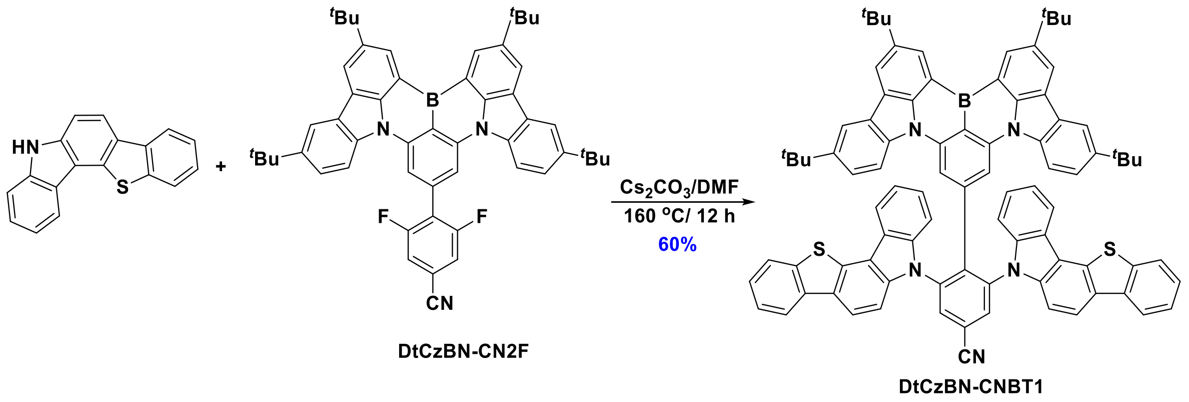

To a 50 mL two-neck flask, were added Cs_2CO_3 (1.3 g, 4 mmol, 4 equiv.), and 5H-benzo[4,5]thieno[3,2-c]carbazole (682 mg, 2.5 mmol, 2 equiv.). The flask was degassed by three cycles of vacuum-nitrogen purging and 10 mL of dry DMF was injected. The mixture was stirred under room temperature for 2 hours and **DtCzBN-CN2F** (770 mg, 1 mmol, 1 equiv.) dissolved in 5 mL of DMF was added. The solution was heated at 160 °C for 12 h and cool down to room temperature. The mixture was washed by saturated NH_4Cl solution (100 mL) and extracted by ethyl acetate (50 mL × 3). The combined organic layers were dried with anhydrous magnesium sulfate. The organic solvent was removed under reduced

pressure and the crude product was purified by silica gel column chromatography. DCM/Hexane=4/1 was used as eluent to afford **DtCzBN-CNBT1** as a yellow solid.

Yield: 60%. **R_f**: 0.27 (DCM). **Mp**: 405 °C. **^{1}H NMR (400 MHz, $CDCl_3$) δ (ppm)**: 9.18 (d, J = 1.7 Hz, 2H), 8.52 (dd, J = 3.4, 1.4 Hz, 2H), 8.37 (dd, J = 7.0, 4.0 Hz, 4H), 8.34 (s, 2H), 8.29 – 8.24 (m, 4H), 7.74 (d, J = 1.2 Hz, 2H), 7.68 – 7.58 (m, 12H), 7.52 (dd, J = 7.2, 1.3 Hz, 4H), 6.03 (d, J = 128.5 Hz, 2H), 1.71 (s, 18H), 1.54 (s, 18H). **^{13}C NMR (101 MHz, $CDCl_3$) δ (ppm)**: 166.6, 166.2, 144.7, 143.4, 141.1, 140.5, 139.9, 138.8, 137.4, 135.6, 133.1, 129.4, 126.5, 124.5, 122.9, 121.1, 120.3, 117.7, 116.6, 115.6, 112.9, 110.0, 107.6, 35.0, 34.3, 32.1, 31.5. **HRMS** (MALDI-TOF) **$[M]^+$ Calculated: ($C_{89}H_{70}BN_5S_2$)** 1283.517; **Found**: 1283.485. **Elemental analysis**: **Calcd for $C_{89}H_{70}BN_5S_2$**: C, 83.22; H, 5.49; N, 5.45. **Found**: C, 83.04; H, 5.44; N, 5.41. **HPLC**: 20% THF/MeOH, 1.0 mL min^{-1}, 300 nm; tr (99.0%) = 7.2 min.

Synthesis of **DtCzBN-CNBT2**

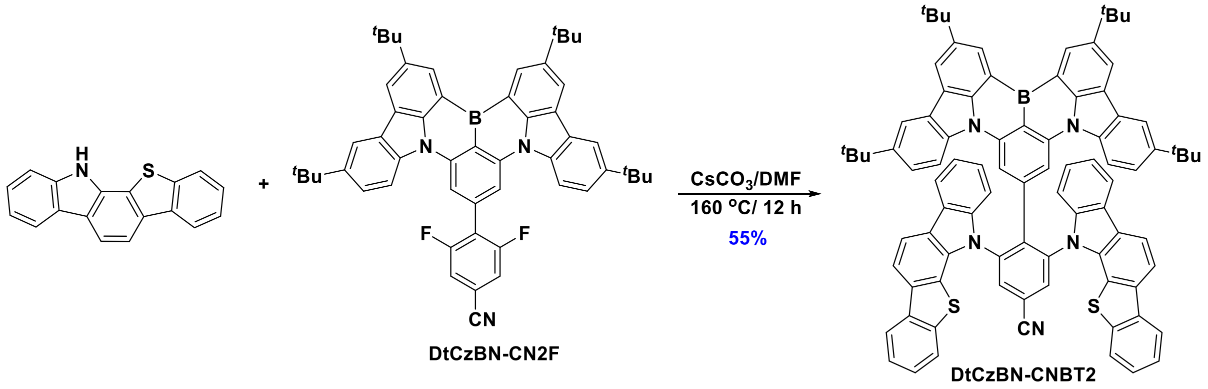

To a 50 mL two-neck flask, were added Cs_2CO_3 (1.3 g, 4 mmol, 4 equiv.), and 12H-benzo[4,5]thieno[2,3-a]carbazole (682 mg, 2.5 mmol, 2 equiv.). The flask was degassed by three cycles of vacuum-nitrogen purging and 10 mL of dry DMF was injected. The mixture was stirred under room temperature for 2 hours and **DtCzBN-CNF** (770 mg, 1 mmol, 1 equiv.) dissolved in 5 mL of DMF was added. The solution was heated at 160 °C for 12 h and cool down to room temperature. The mixture was washed by saturated NH_4Cl solution (100 mL) and extracted by ethyl acetate (50 mL × 3). The combined organic layers were dried with anhydrous magnesium sulfate. The organic solvent was

removed under reduced pressure and the crude product was purified by silica gel column chromatography. DCM/Hexane=4/1 was used as eluent to afford **DtCzBN-CNBT2** as a yellow solid.

Yield: 55%. **R_f**: 0.26 (DCM). **Mp**: 403 °C. **^{1}H NMR (400 MHz, $CDCl_3$) δ (ppm)**: 9.17 (d, *J* = 1.7 Hz, 2H), 8.51 (dd, *J* = 3.4, 1.4 Hz, 2H), 8.48 – 8.39 (m, 4H), 8.36 (s, 2H), 8.33 – 8.14 (m, 4H), 7.74 (d, *J* = 1.2 Hz, 2H), 7.71 – 7.53 (m, 12H), 7.47 (dd, *J* = 7.2, 1.3 Hz, 4H), 6.03 (d, *J* = 120.1 Hz, 2H), 1.70(s, 18H), 1.54 (s, 18H). **^{13}C NMR (101 MHz, $CDCl_3$) δ (ppm)**: 168.9, 167.6, 145.5, 144.8, 144.2, 142.6, 141.7, 141.1, 140.4, 138.3, 136.9, 134.0, 129.9, 127.1, 124.5, 123.8, 121.7, 120.9, 120. 8, 119.6, 117.3, 114.1, 110.9, 109.3, 35.2, 34.8, 32.2, 31.8. **HRMS** (MALDI-TOF) **$[M]^+$ Calculated: ($C_{89}H_{70}BN_5S_2$)** 1283.517; **Found**: 1283.509. **Elemental analysis**: **Calcd for $C_{89}H_{70}BN_5S_2$**: C, 83.22; H, 5.49; N, 5.45. **Found**: C, 83.69; H, 5.53; N, 5.48. **HPLC**: 20% THF/MeOH, 1.0 mL min^{-1}, 300 nm; tr (99.0%) = 7.2 min.

Synthesis of **CNBT1**

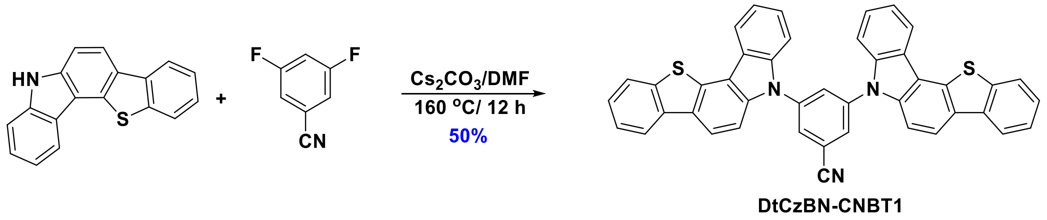

To a 50 mL two-neck flask, were added Cs_2CO_3 (700 mg, 2 mmol, 4 equiv.), and 5H-benzo[4,5]thieno[3,2-c]carbazole (380 mg, 1.1 mmol, 2.2 equiv.). The flask was degassed by three cycles of vacuum-nitrogen purging and 5 mL of dry DMF was injected. The mixture was stirred at 80 °C for 2 hours and 3,5-difluorobenzonitrile (280 mg, 0.5 mmol, 1 equiv.) dissolved in 5 mL of DMF was then added. The solution was heated at 160 °C for 12 h and then cooled to room temperature. The mixture was washed with saturated NH_4Cl solution (100 mL) and extracted with ethyl acetate (50 mL × 3). The combined organic layers were dried with anhydrous magnesium sulfate. The organic solvent was removed under reduced pressure and the crude product was purified by silica gel column chromatography (DCM/Hexane=4/1 was used as the eluent) to afford **CNBT1** as a white solid.

Yield: 50%. **R$_f$**: 0.25 (DCM). **Mp**: 205 °C. **^{1}H NMR (400 MHz, CDCl$_3$) δ (ppm)**: 8.35 (d, *J* = 7.6 Hz, 2H), 8.30 – 8.16 (m, 6H), 8.13 (s, 1H), 8.01 (d, *J* = 7.7 Hz, 2H), 7.75 – 7.62 (m, 4H), 7.61 – 7.42 (m, 8H). **^{13}C NMR (101 MHz, CDCl$_3$) δ (ppm)**: 140.8, 139.9, 139.4, 138.8, 135.6, 133.2, 130.2, 129.8, 129.4, 129.0, 126.4, 125.8, 124.8, 123.1, 122.2, 121.9, 121.2, 119.9, 119.5, 117.9, 116.0, 109.4, 106.9. **HRMS** (ESI-MS) **[M]$^+$ Calculated: ($C_{43}H_{23}N_3S_2$)** 645.7980; **Found**: 645.6196.

Synthesis of **CNBT2**

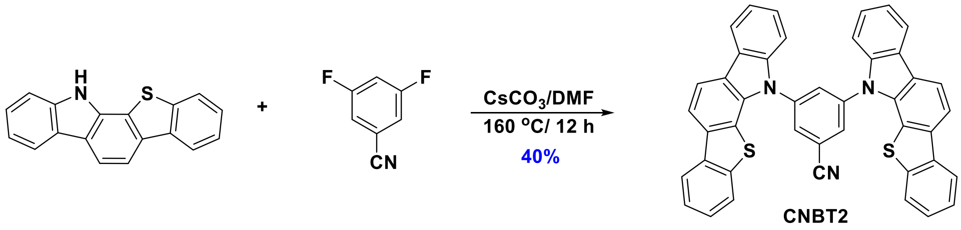

To a 50 mL two-neck flask, were added Cs_2CO_3 (700 mg, 2 mmol, 4 equiv.), and 12H-benzo[4,5]thieno[2,3-a]carbazole (280 mg, 1.1 mmol, 2.2 equiv.). The flask was degassed by three cycles of vacuum-nitrogen purging and 10 mL of dry DMF was injected. The mixture was stirred at 80 °C for 2 hours and 3,5-difluorobenzonitrile (280 mg, 0.5 mmol, 1 equiv.) dissolved in 5 mL of DMF was added. The solution was heated at 160 °C for 12 h and then cooled to room temperature. The mixture was washed with saturated NH_4Cl solution (100 mL) and extracted with ethyl acetate (50 mL × 3). The combined organic layers were dried with anhydrous magnesium sulfate. The organic solvent was removed under reduced pressure and the crude product was purified by silica gel column chromatography (DCM/Hexane=4/1 was used as then eluent) to afford **CNBT2** as a white solid.

Yield: 40%. **R$_f$**: 0.29 (DCM). **Mp**: 189 °C. **^{1}H NMR (400 MHz, CDCl$_3$) δ (ppm)**: 8.36 – 8.31 (m, 2H), 8.24 (td, *J* = 5.0, 2.6 Hz, 4H), 8.10 (d, *J* = 1.6 Hz, 1H), 8.01 (d, *J* = 5.9 Hz, 2H), 7.71 – 7.65 (m, 2H), 7.63 (d, *J* = 6.9 Hz, 4H), 7.60 – 7.47 (m, 8H). **^{13}C NMR (101 MHz, CDCl$_3$) δ (ppm)**: 141.9, 141.2, 139.3, 135.7, 135.2, 133.3, 132.2, 130.6, 129.7, 126.6, 126.0, 124.9, 124.6, 122.7, 121.7, 120.7, 120.6, 117.5, 114.8, 114.1, 110.3, 109.9, 101.7. **HRMS** (ESI-MS) **[M]$^+$ Calculated: ($C_{43}H_{23}N_3S_2$)** 645.7980; **Found**: 645.6025.

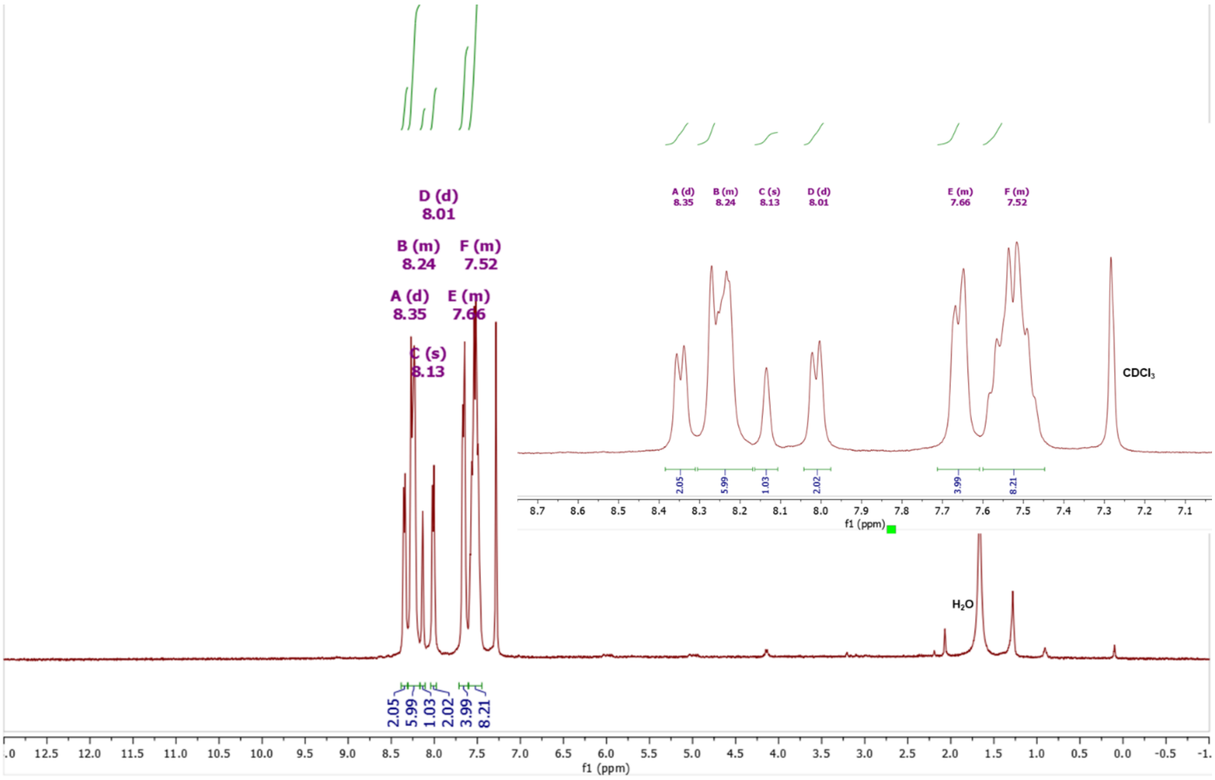

Figure **S1**. ^{1}H NMR spectrum of **CNBT1** in $CDCl_3$.

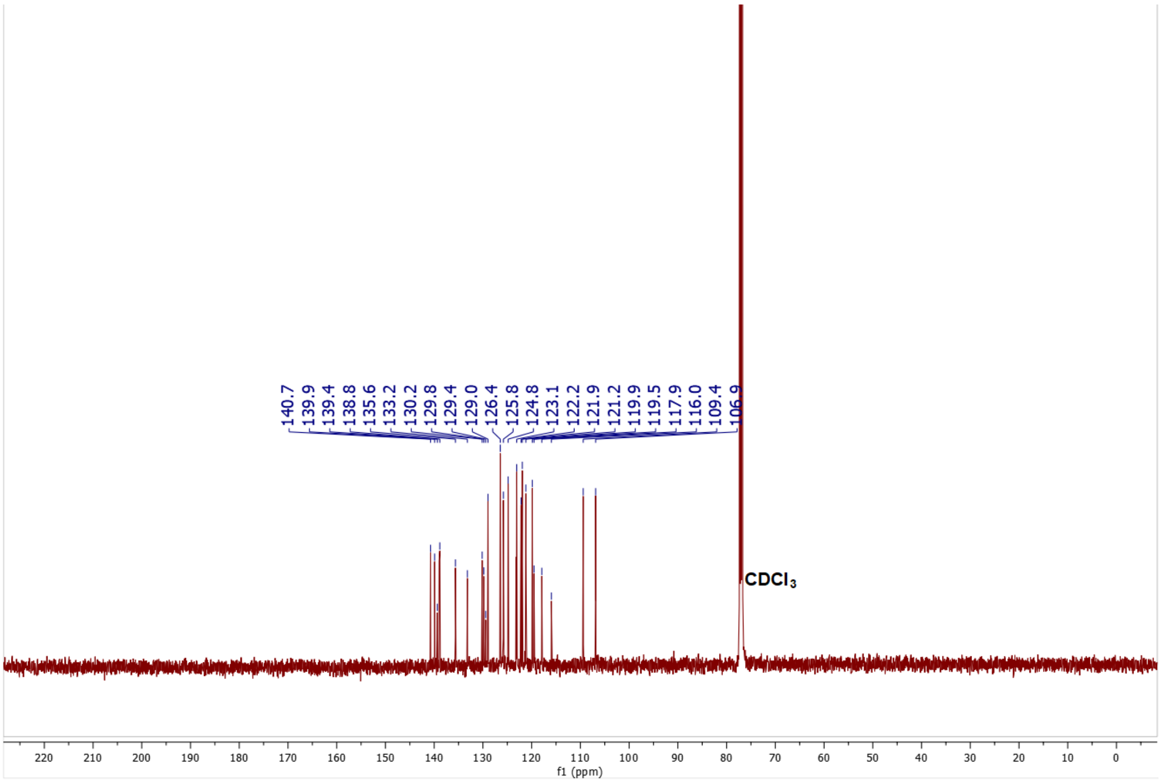

Figure **S2**. ^{13}C NMR spectrum of **CNBT1** in $CDCl_3$.

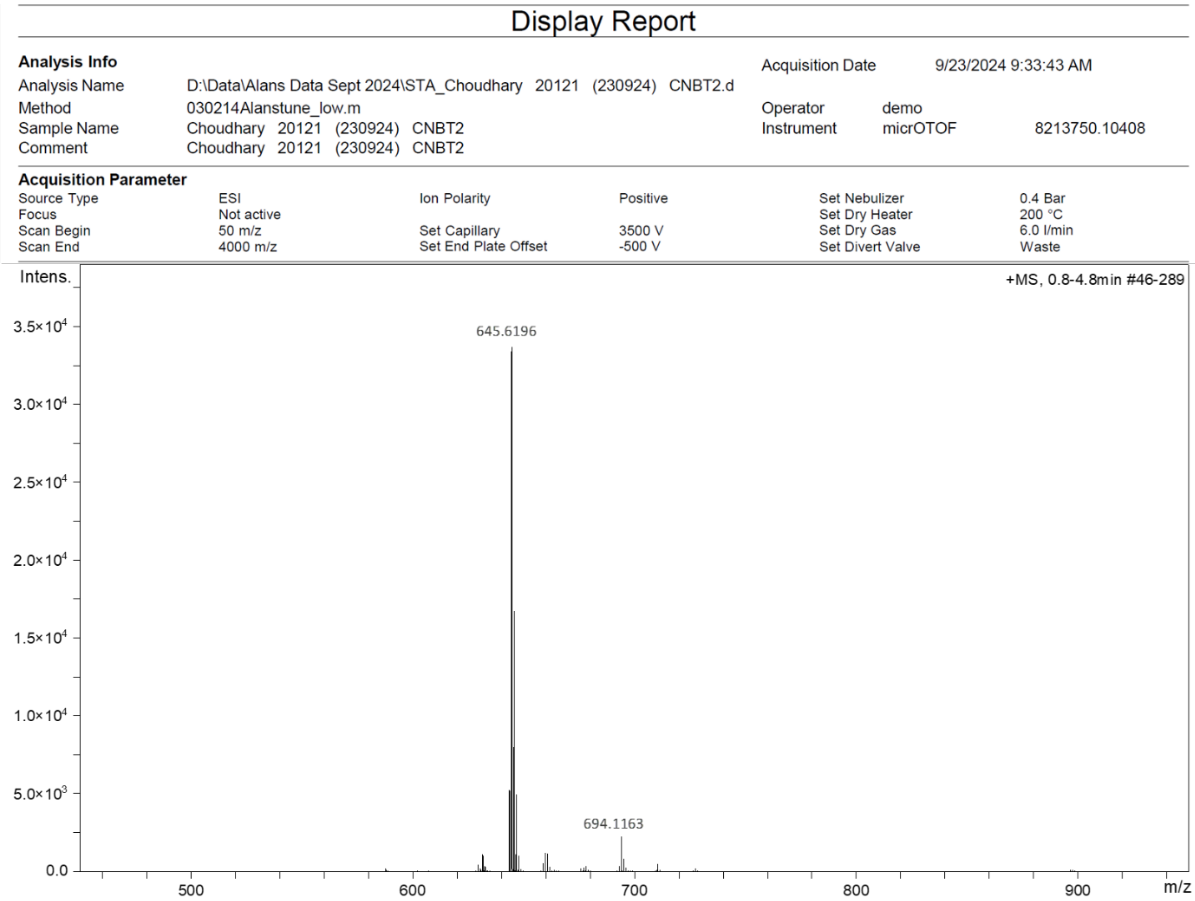

Figure **S3**. HRMS of **CNBT1**.

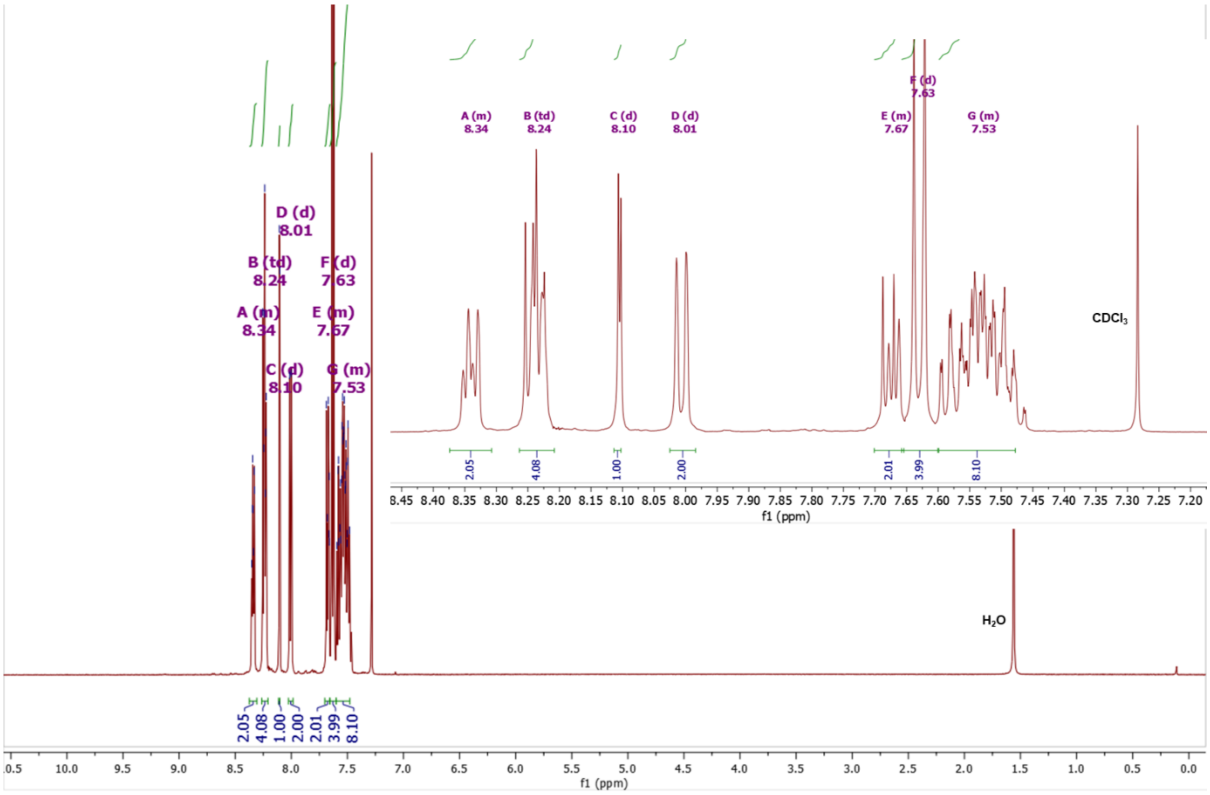

Figure **S4**. ^{1}H NMR spectrum of **CNBT2** in $CDCl_3$.

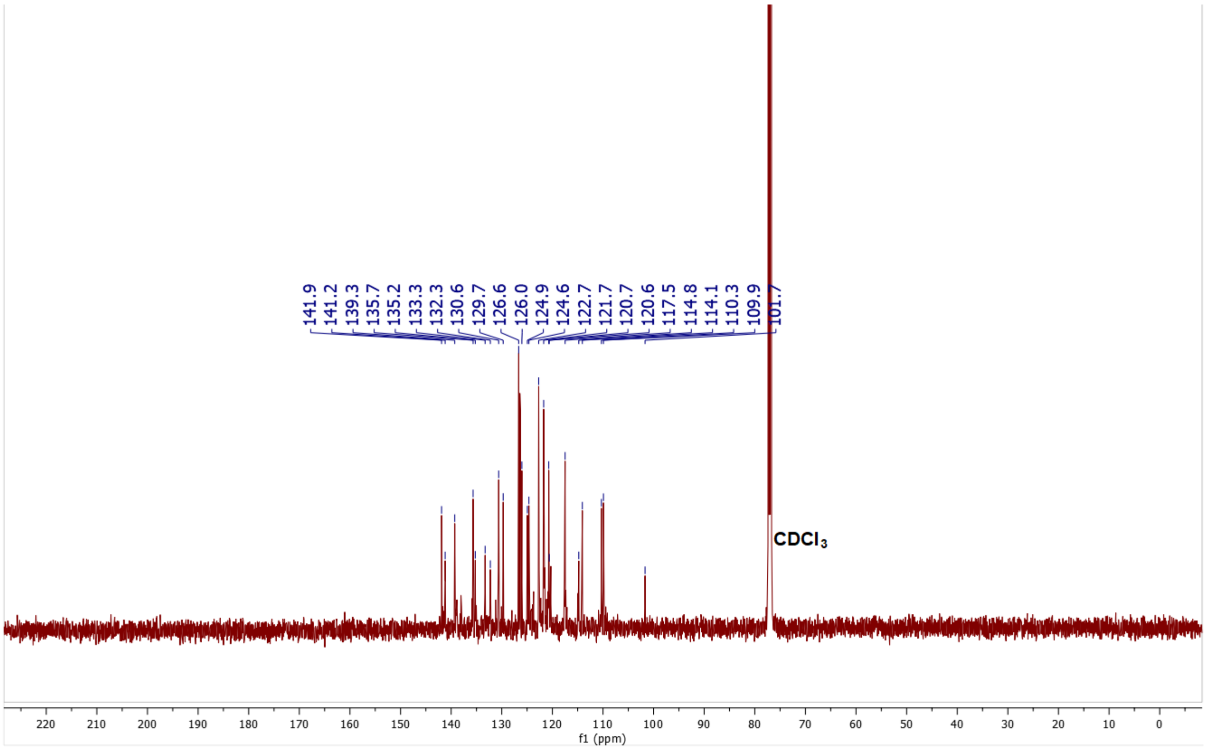

Figure **S5**. ^{13}C NMR spectrum of **CNBT2** in $CDCl_3$.

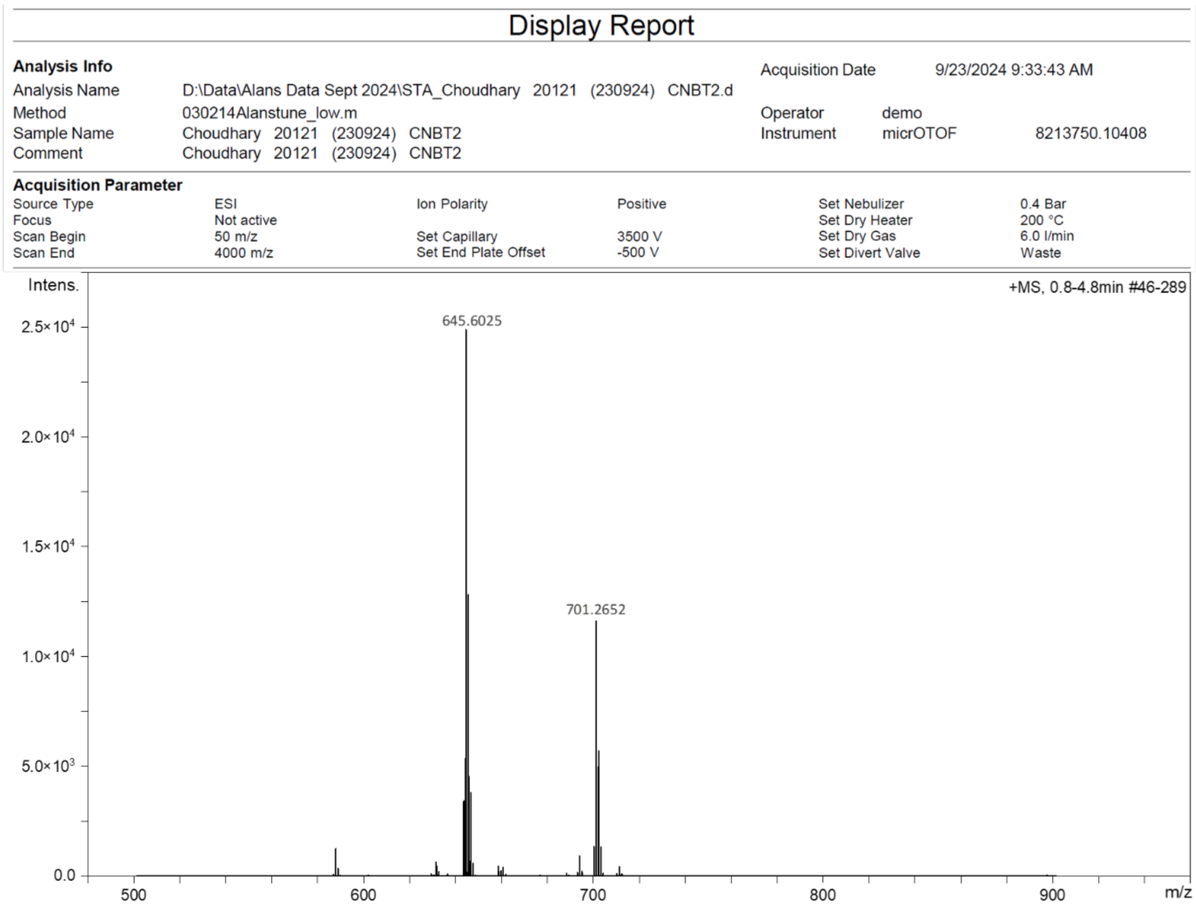

Figure **S6**. HRMS of **CNBT2**.

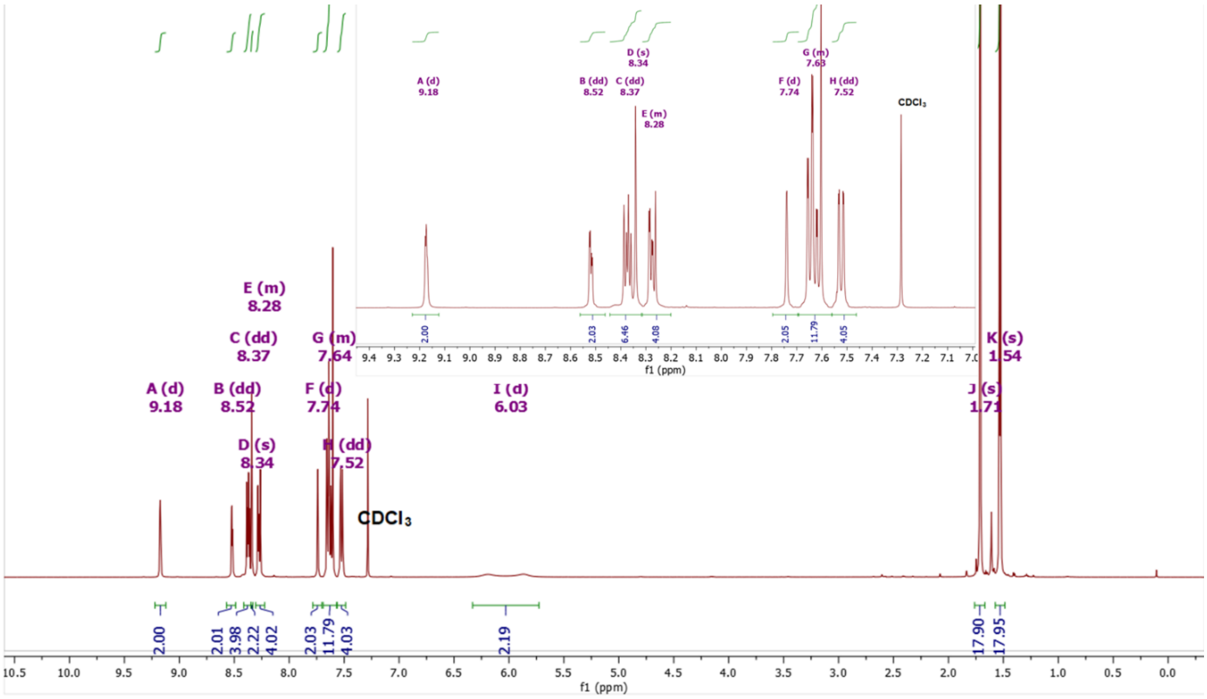

Figure **S7**. ^{1}H NMR spectrum of **DtCzBN-CNBT1** in $CDCl_3$.

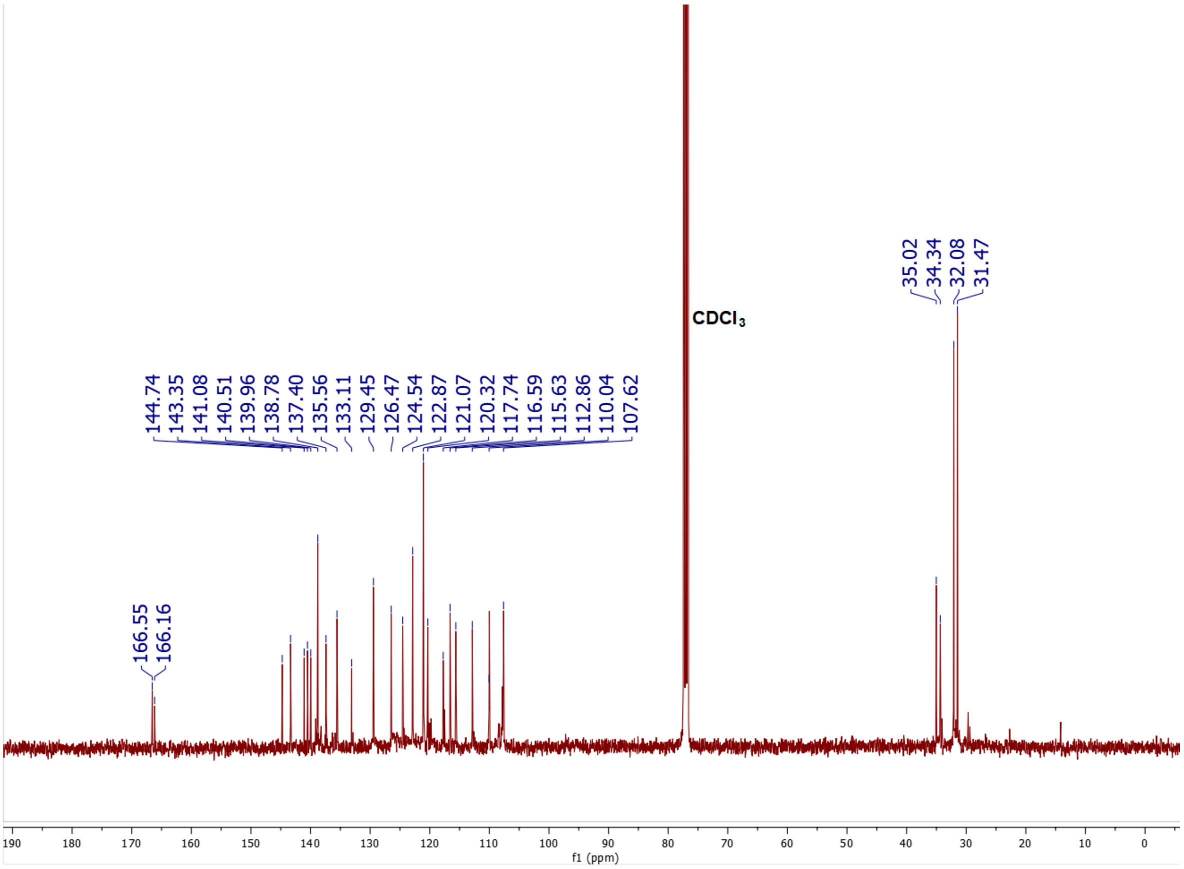

Figure **S8**. ^{13}C NMR spectrum of **DtCzBN-CNBT1** in $CDCl_3$.

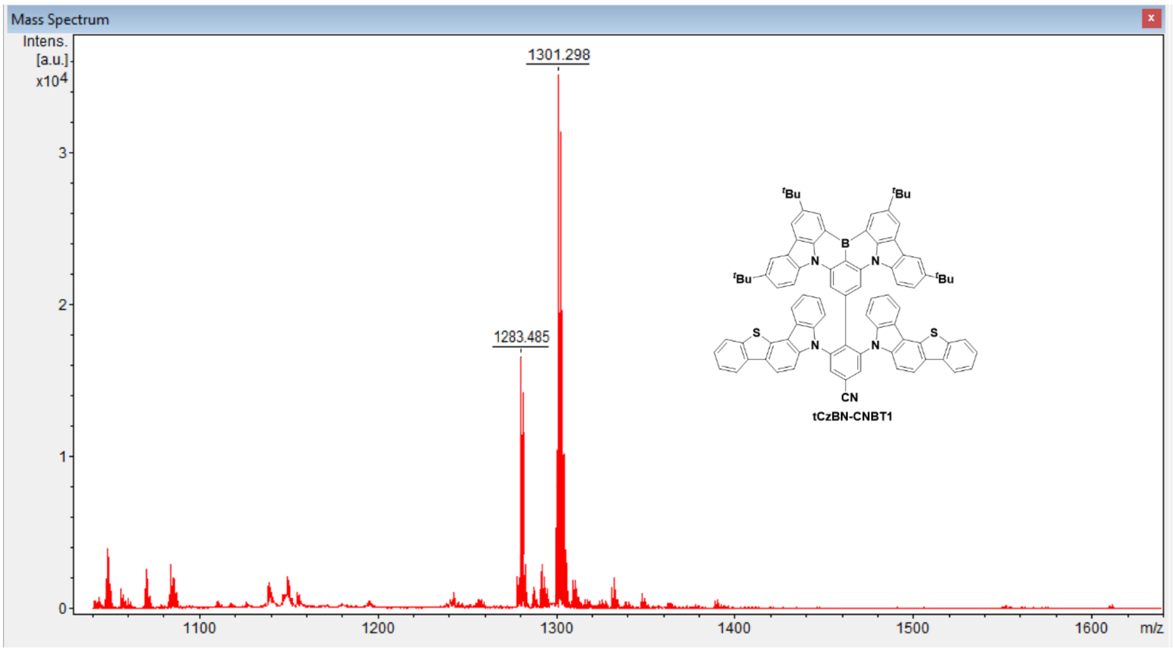

Figure **S9**. MALDI-TOF spectra of **DtCzBN-CNBT1**.

Elemental Analysis Service Request Form

Researcher name Dongyang Chen

Researcher email dc217@st-andrews.ac.uk

NOTE: Please submit ca. 10 mg of sample

**Sample reference number**	DC-IV040723
**Name of Compound**	tCzB-CNBT1
**Molecular formula**	C89H70BN5S2
**Stability**	Stable
**Hazards**	
**Other Remarks**	

Analysis type:

Single ☐ **Duplicate** ■ **Triplicate** ☐

Analysis Result:

Element	Expected %	Found (1)	Found (2)	Found (3)
Nitrogen	5.45	5.40	5.42	
Carbon	83.22	82.98	83.11	
Hydrogen	5.49	5.44	5.43	

Authorising Signature:

**Date completed**	09.05.23
**Signature**	[illegible]
**comments**	

Figure **S10**. Elemental Analysis of **DtCzBN-CNBT1**.

HPLC Trace Report30Jul2023

<Sample Information>

Sample Name	: tCzB-CNBT1		
Sample ID	: dc-TCzBCNBT1-001		
Method Filename	: 99% Methanol 1% THF 20 mins		
Batch Filename	: dc-tCzB-CN		
Vial #	: 1-53	Sample Type	: Unknown
Injection Volume	: 10 uL		
Date Acquired	: 30/05/2023 15:33:31	Acquired by	: System Administrator
Date Processed	: 30/05/2023 15:53:33	Processed by	: System Administrator

<Chromatogram>

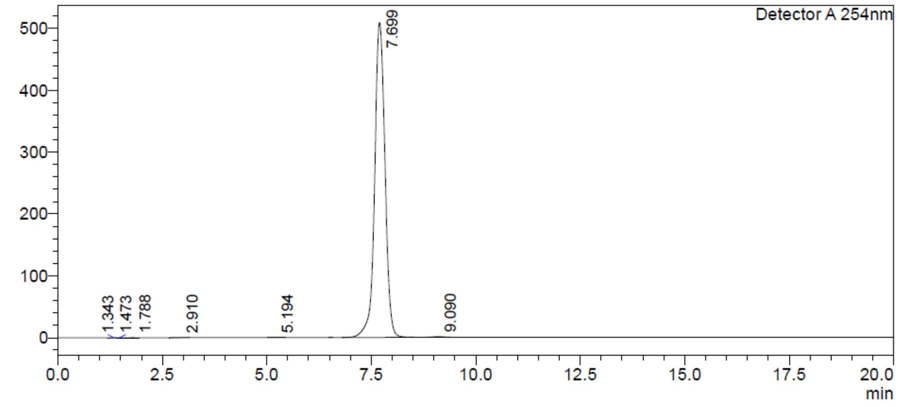

<Peak Table>

Detector A 254nm

Peak#	Ret. Time	Area	Height	Area%	Area/Height	Width at 5% Height
1	1.343	1925	257	0.022	7.481	--
2	1.473	1045	177	0.012	5.910	--
3	1.788	2643	156	0.030	16.982	--
4	2.910	3133	280	0.036	11.202	0.341
5	5.194	1495	126	0.017	11.836	0.349
6	7.699	8740085	508263	99.661	17.196	0.603
7	9.090	19469	1025	0.222	18.998	0.600
Total		8769795	510284	100.000		

Figure **S11**. HPLC report of **DtCzBN-CNBT1**.

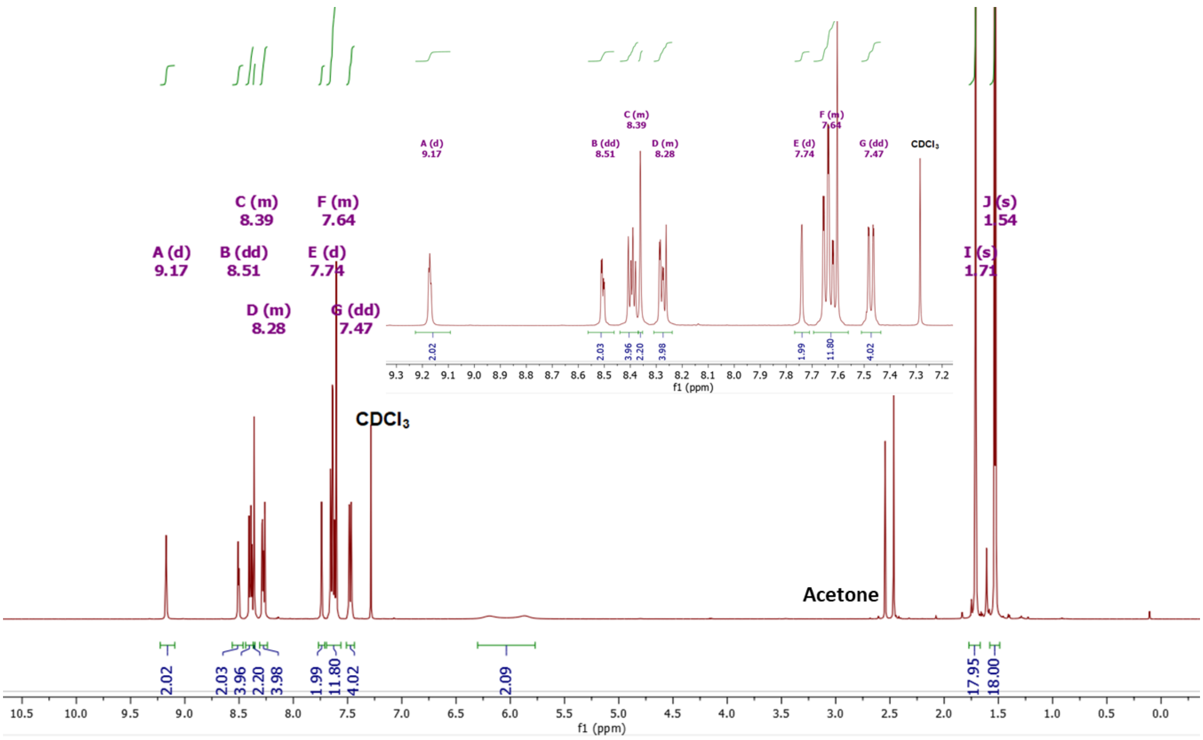

Figure **S12**. ^{1}H NMR spectrum of **DtCzBN-CNBT2** in $CDCl_3$.

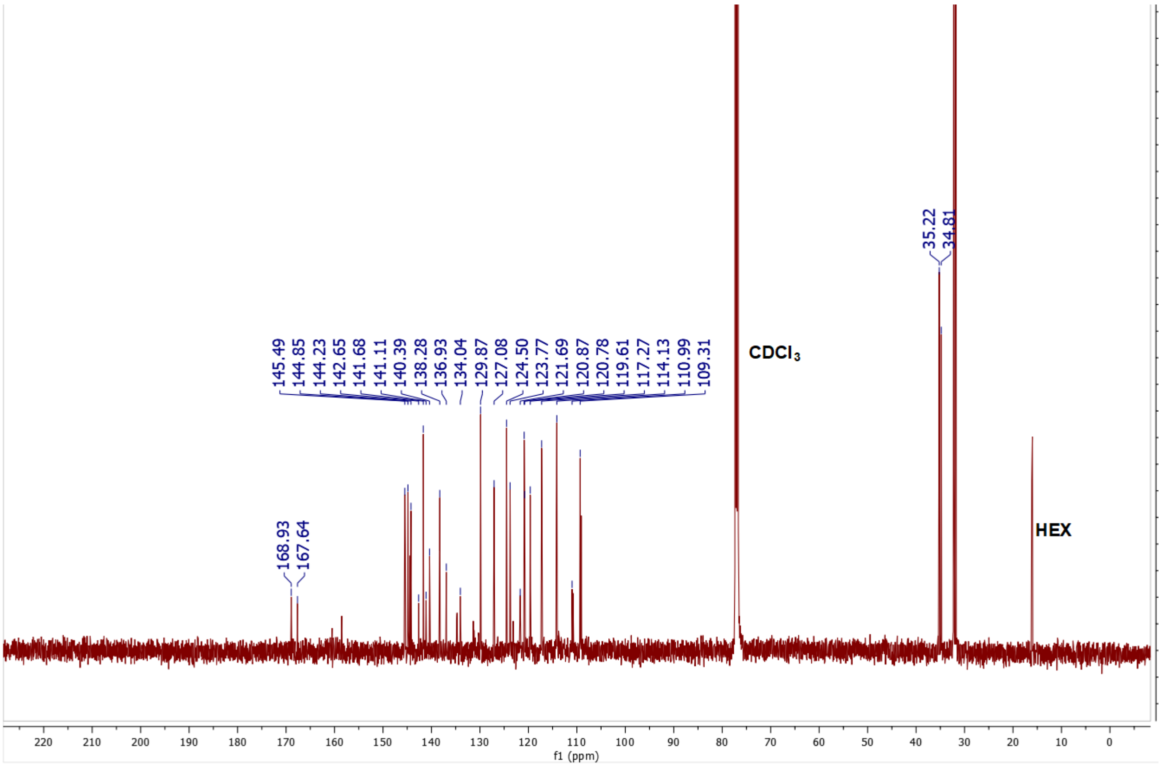

Figure **S13**. ^{13}C NMR spectrum of **DtCzBN-CNBT2** in $CDCl_3$.

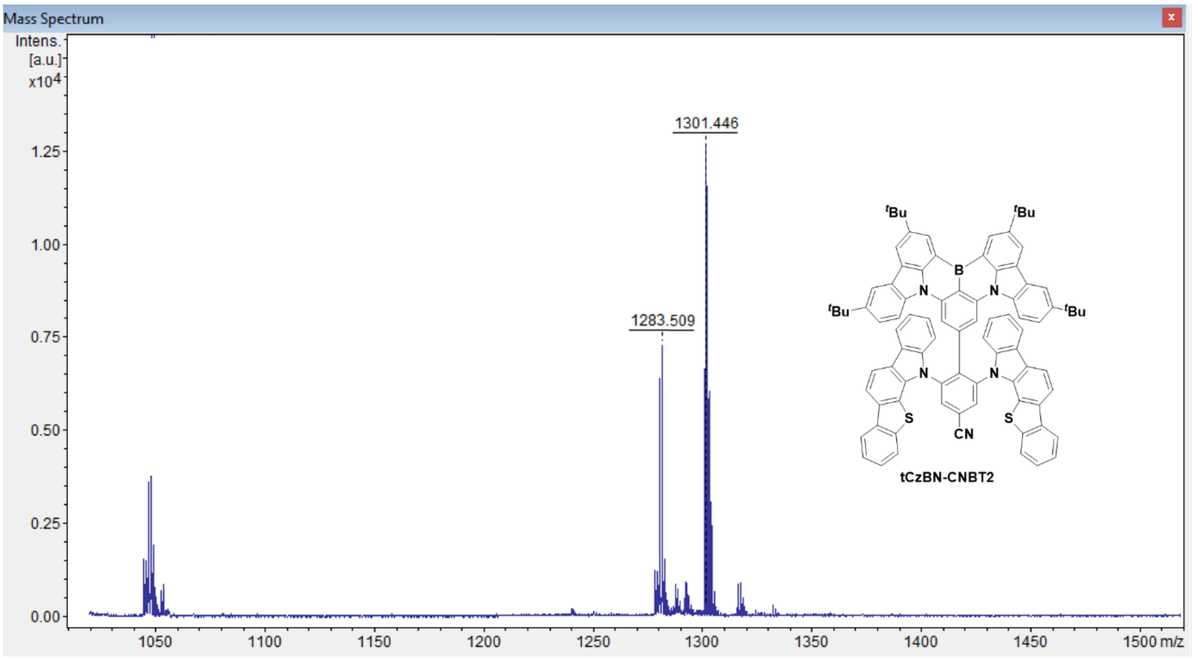

Figure **S14**. MALDI-TOF spectra of **DtCzBN-CNBT2**.

Elemental Analysis Service Request Form

Researcher name Dongyang Chen

Researcher email dc217@st-andrews.ac.uk

NOTE: Please submit ca. 10 mg of sample

**Sample reference number**	DC-IV141122
**Name of Compound**	tCzB-CNBT2
**Molecular formula**	C89H70BN5S2
**Stability**	Stable
**Hazards**	
**Other Remarks**	

Analysis type:

Single ☐ **Duplicate** ■ **Triplicate** ☐

Analysis Result:

Element	Expected %	Found (1)	Found (2)	Found (3)
Nitrogen	5.45	5.47	5.49	
Carbon	83.22	83.68	83.71	
Hydrogen	5.49	5.51	5.54	

Authorising Signature:

**Date completed**	07.05.23
**Signature**	[illegible]
**comments**	

Figure **S15**. Elemental Analysis of **DtCzBN-CNBT2**.

HPLC Trace Report30Jul2023

<Sample Information>

Sample Name	: dc-tCzB-CNBT2-2		
Sample ID	:		
Method Filename	: 99% Methanol 1% THF 20 mins		
Batch Filename	: 11072023.lcb		
Vial #	: 1-50	Sample Type	: Unknown
Injection Volume	: 10 uL		
Date Acquired	: 11/07/2023 16:14:00	Acquired by	: System Administrator
Date Processed	: 11/07/2023 16:34:03	Processed by	: System Administrator

<Chromatogram>

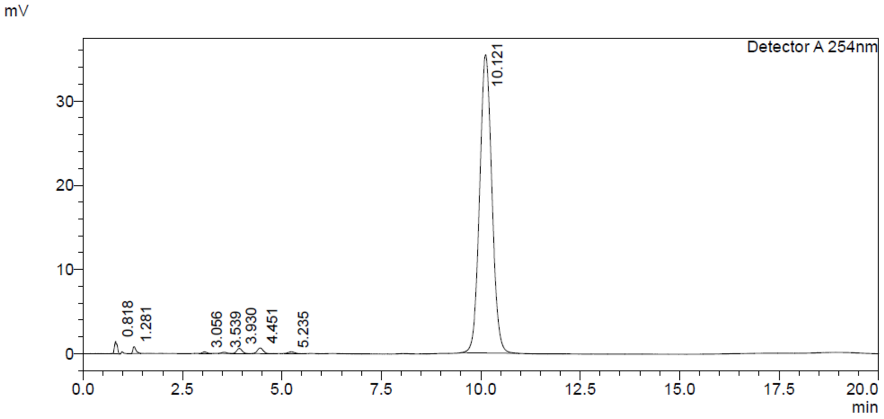

<Peak Table>

Detector A 254nm

Peak#	Ret. Time	Area	Height	Area%	Area/Height	Width at 5% Height
1	0.818	6884	1424	0.872	4.835	0.134
2	1.281	4374	818	0.554	5.346	0.190
3	3.056	1449	189	0.184	7.666	0.224
4	3.539	1213	148	0.154	8.218	0.239
5	3.930	5778	608	0.732	9.504	0.305
6	4.451	7096	680	0.899	10.438	0.342
7	5.235	2149	205	0.272	10.480	0.299
8	10.121	760265	35411	96.333	21.470	0.719
Total		789208	39483	100.000		

Figure **S16**. HPLC report of **DtCzBN-CNBT2**.

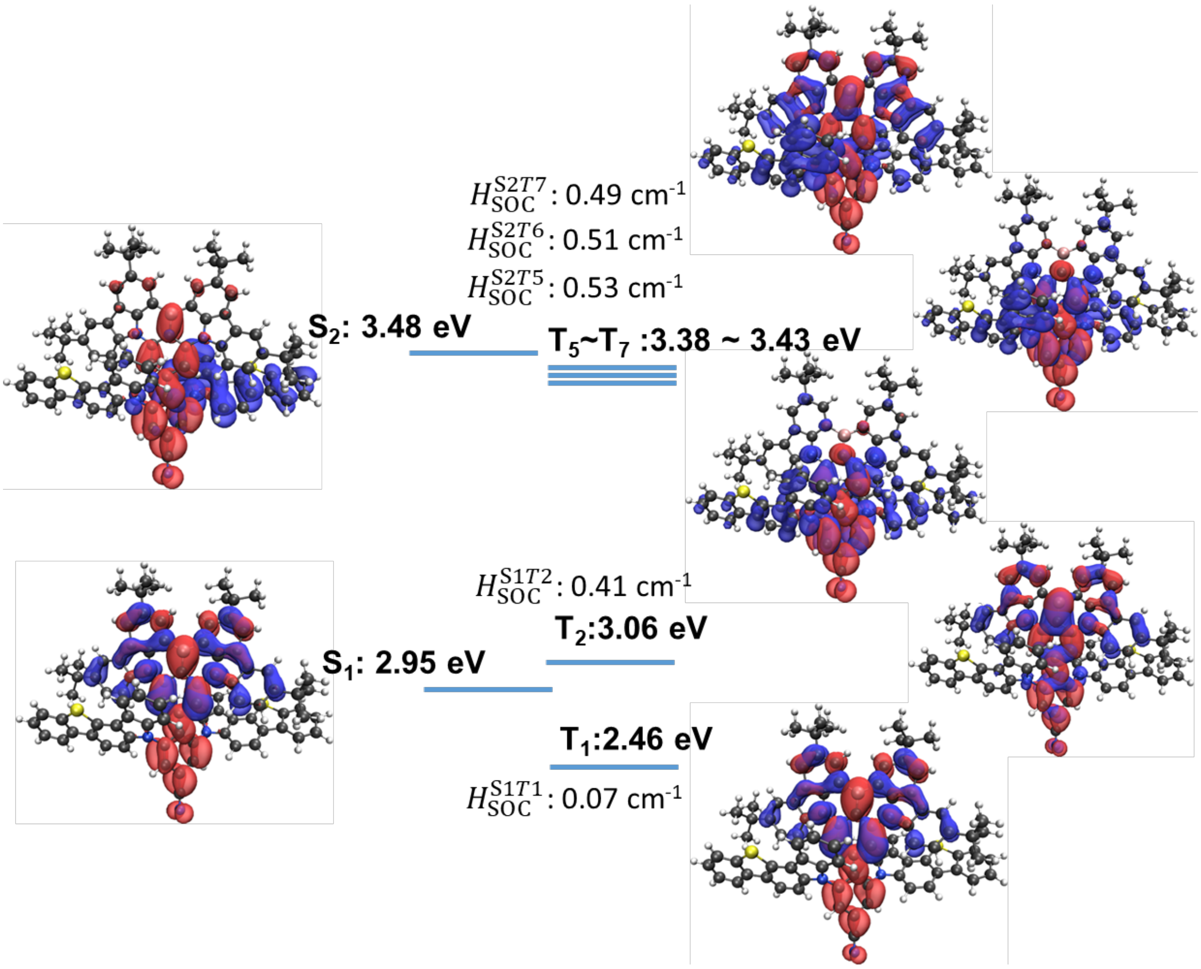

Figure **S17**. Calculated energies and SOCME values between higher-lying excited states of **DtCzBN-CNBT1** based on the optimized T_1 geometry (blue: HONTO, red: LUNTO, isovalue: 0.02).

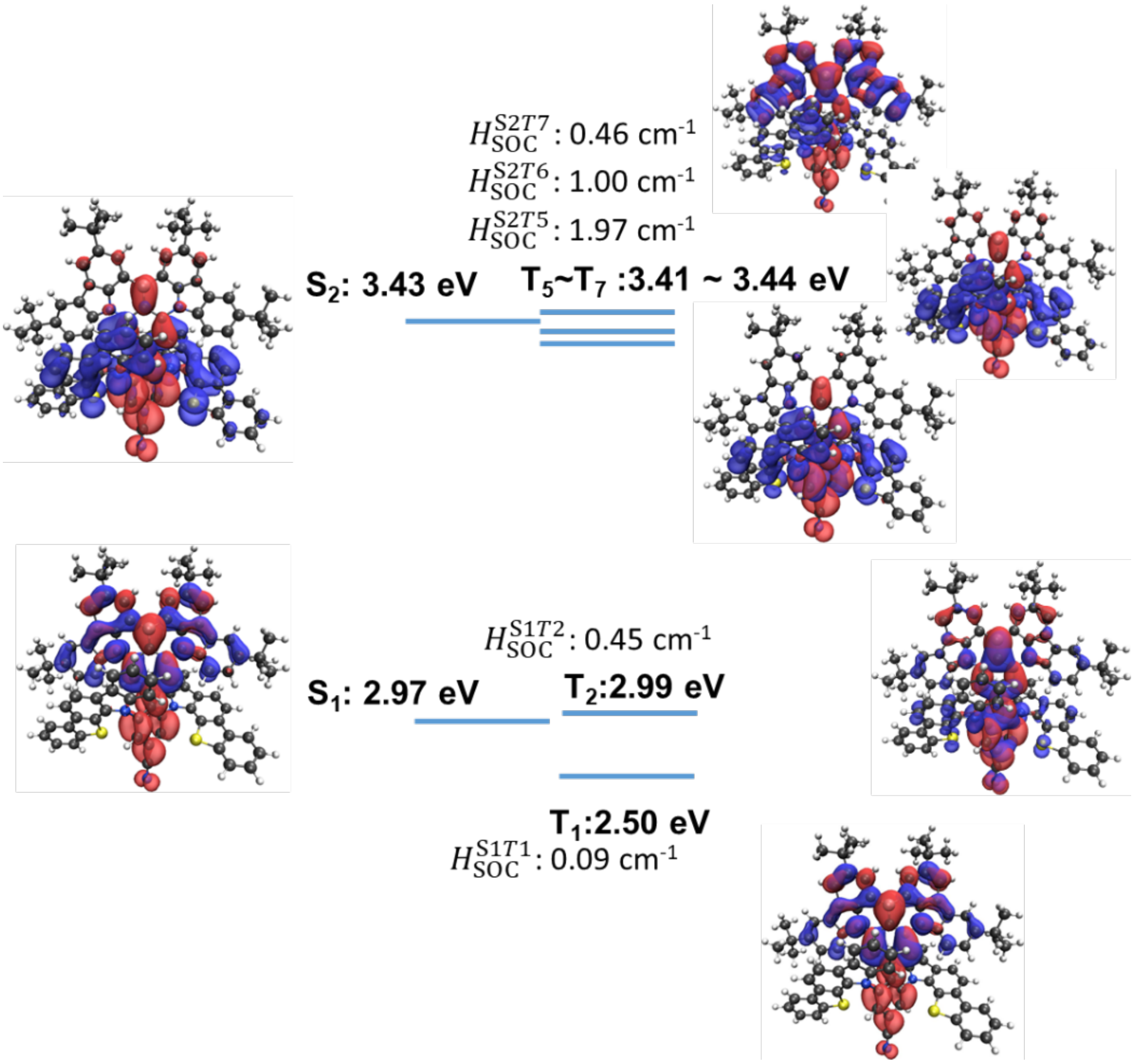

Figure **S18**. Calculated energies of and SOCME values between higher-lying excited states of **DtCzBN-CNBT2** based on the optimized T_1 geometry (blue: HONTO, red: LUNTO, isovalue: 0.02).

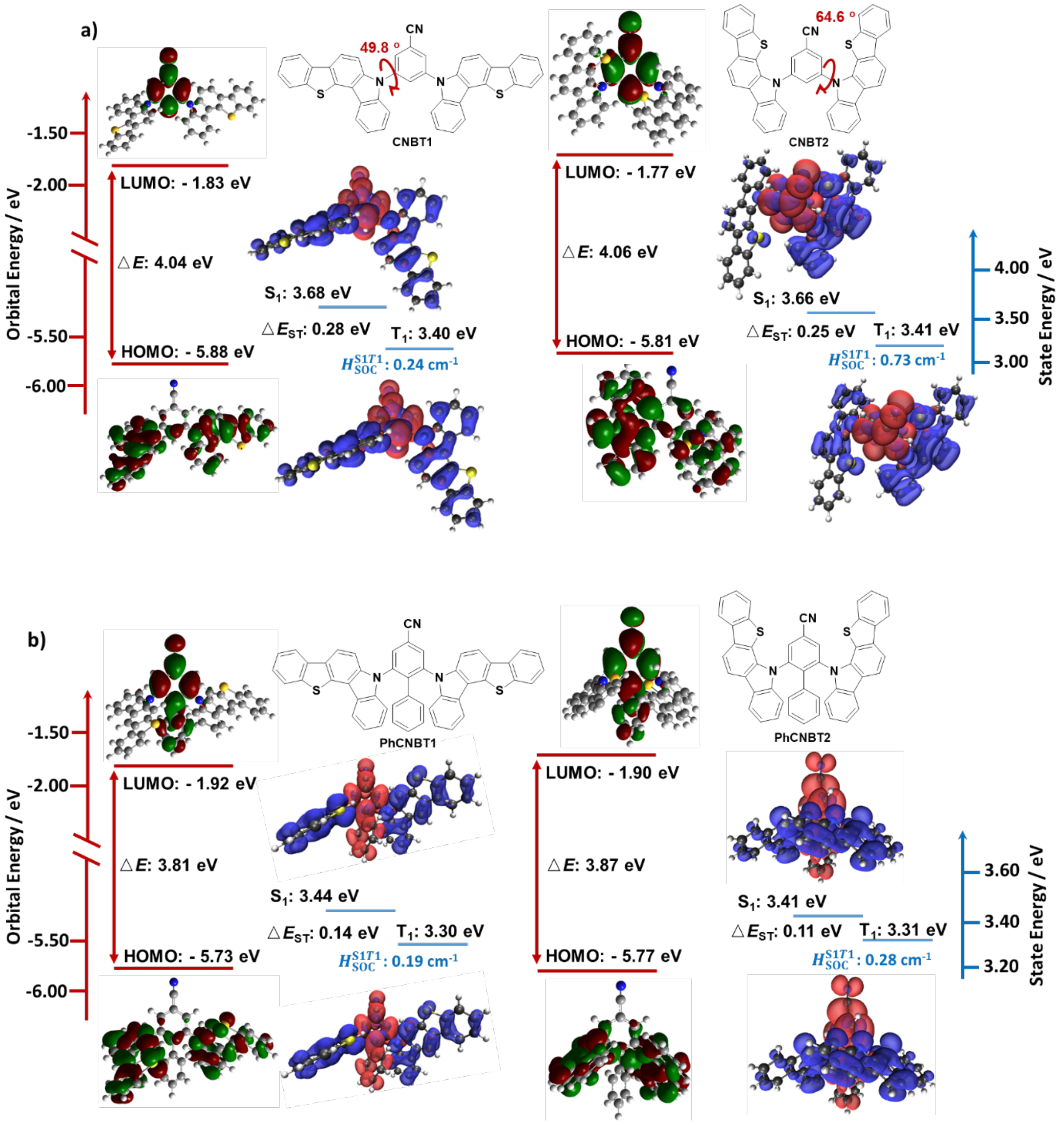

Figure **S19**. Calculated energies of and SOCME values between S_1 and T_1 excited states of a) **CNBT1** and **CNBT2**, and b) **PhCNBT1** and **PhCNBT2**.

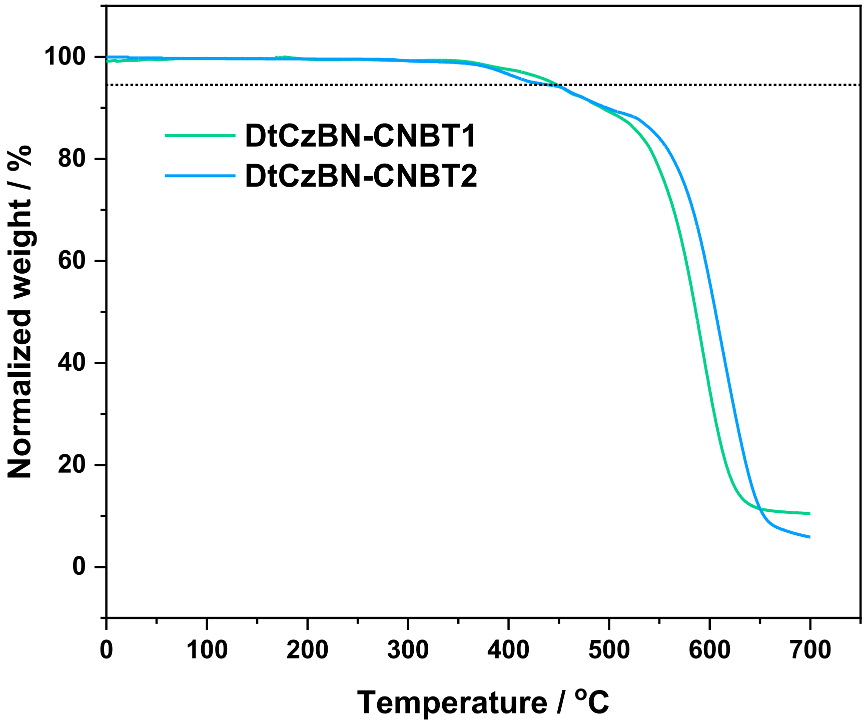

Figure **S20**. TGA analysis of **DtCzBN-CNBT1** and **DtCzBN-CNBT2** under a N_2 atmosphere (heating speed: 5 °C/ min. The dashed line represents the threshold for a 5 wt% weight loss).

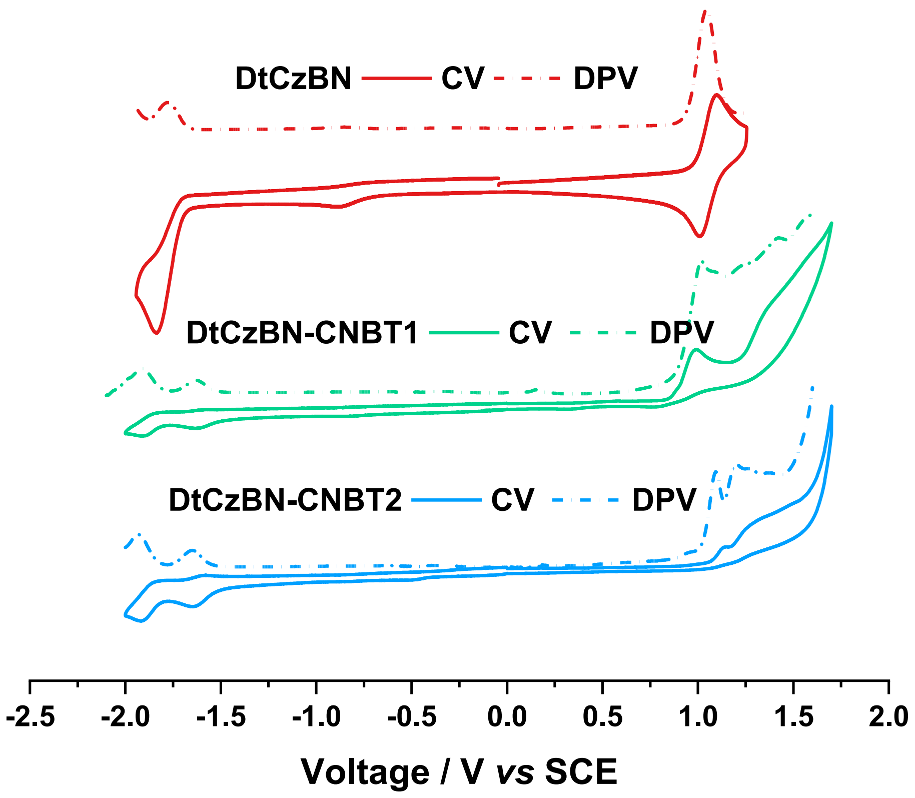

Figure **S21**. Cyclic voltammogram (CV) and differential pulse voltammetry (DPV) for **DtCzBN-CNBT1** and **DtCzBN-CNBT2** in degassed DMF with 0.1 M $[^{n}Bu_4N]PF_6$ as the supporting electrolyte and Fc/Fc^+ as the internal reference versus SCE (0.45 V *vs*. DMF) (Scan rate: 100 mV/s, direction: negative).

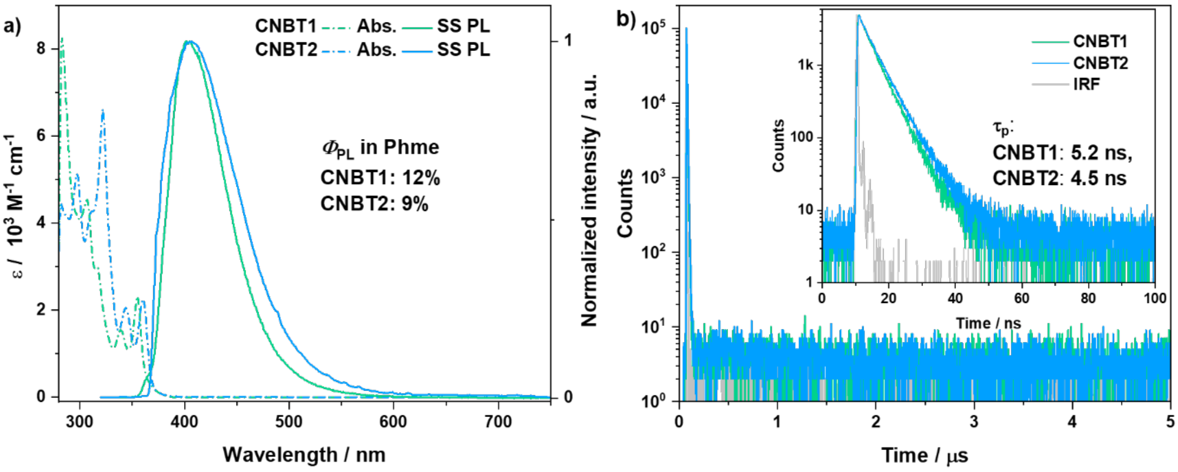

Figure **S22**. a) Absorption and steady-state PL spectra (SS PL) of **CNBT1** and **CNBT2** in dilute toluene at room temperature (λ_{exc} = 300 nm); b) Time-resolved PL decays of **CNBT1** and **CNBT2** in dilute toluene, within a 100 ns window (inset) and 5 μs window (λ_{exc} = 379 nm).

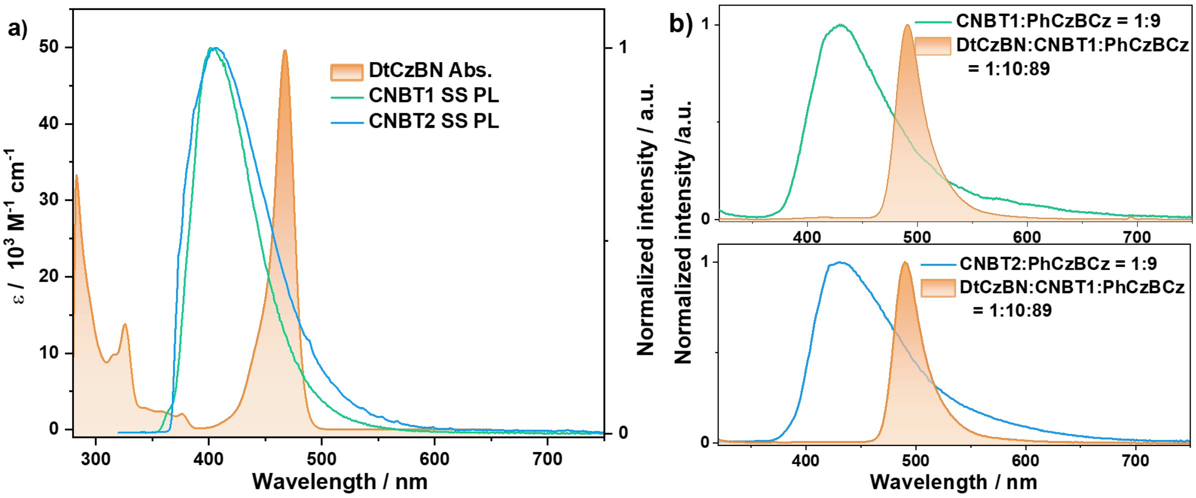

Figure **S23**. a) The spectral overlap between the absorption of **DtCzBN** *versus* the PL of **CNBT1** and **CNBT2** in dilute toluene (λ_{exc} = 300 nm), b) SS PL spectra comparison of the 10 wt% doped films of **CNBT1** or **CNBT2** in PhCzBCz *versus* the 1 wt% **DtCzBN** : 10 wt% **CNBT1** or **CNBT2** : 89 wt% PhCzBCz for FRET efficiency analysis (λ_{exc} = 300 nm).

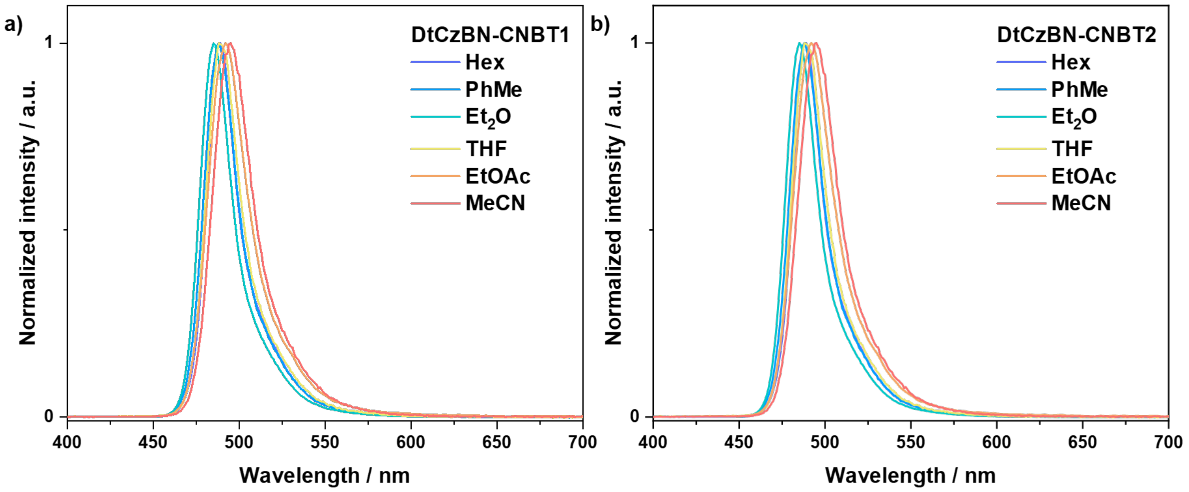

Figure **S24**. Solvatochromism PL study for **DtCzBN-CNBT1** and **DtCzBN-CNBT2** (λ_{exc} = 340 nm).

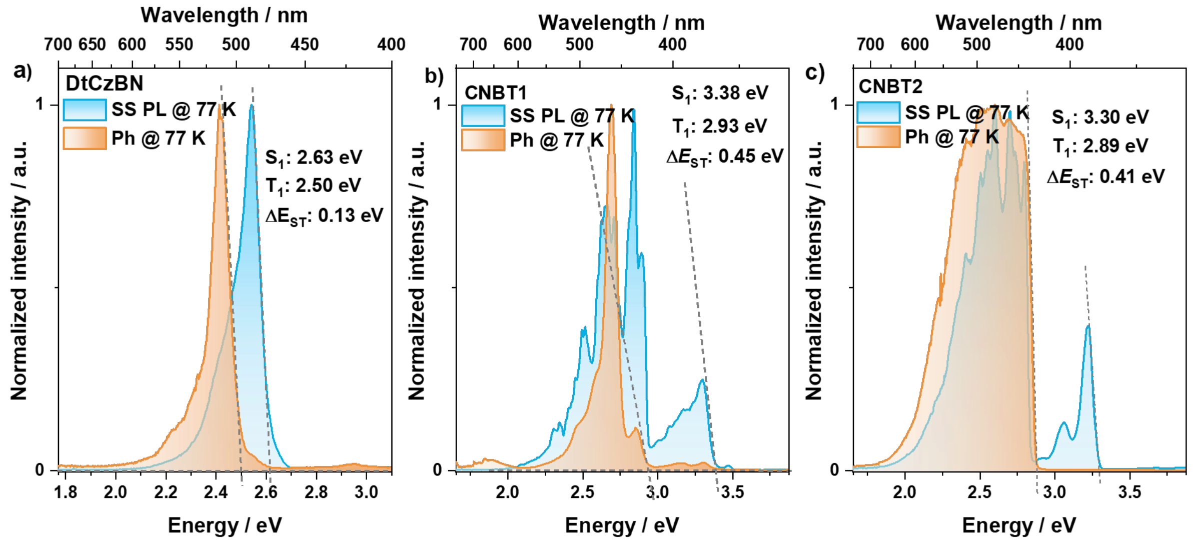

Figure **S25**. Steady-state PL and phosphorescence spectra (1–10 ms) at 77 K in frozen 2-MeTHF for **DtCzBN**, **CNBT1**, and **CNBT2** (λ_{exc} = 340 nm, S_1 and T_1 calculated from the onset of the SS PL and Ph spectra).

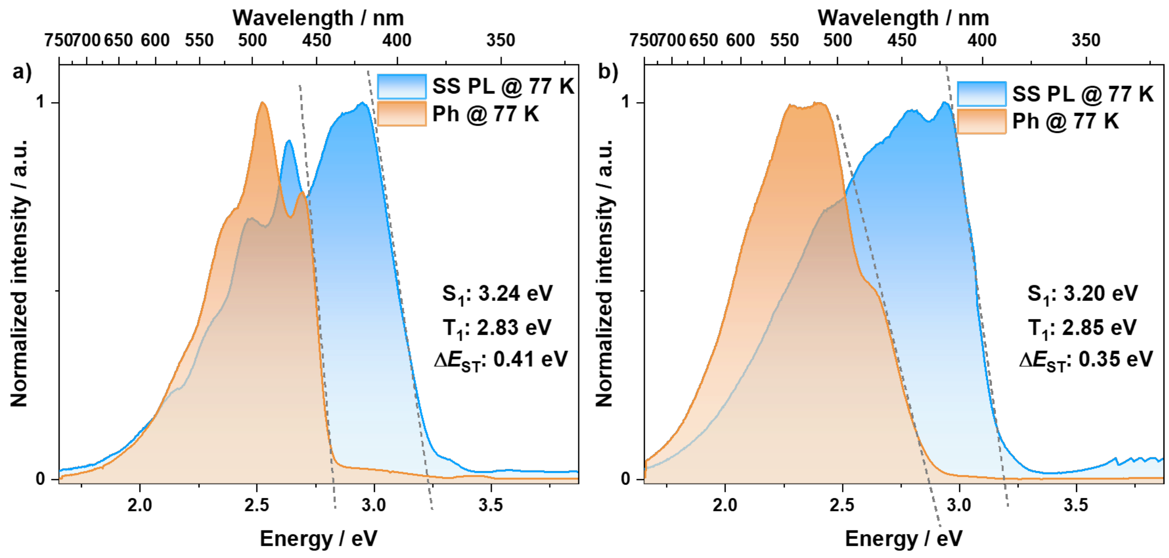

Figure **S26**. Steady-state PL and time-gated phosphorescence spectra (4–20 ms) for a) **CNBT1**, and b) **CNBT2** at 77 K in 15 wt% doped PhCzBCz film (λ_{exc} = 340 nm, S_1 and T_1 calculated from the onset of the SS PL and Ph spectra).

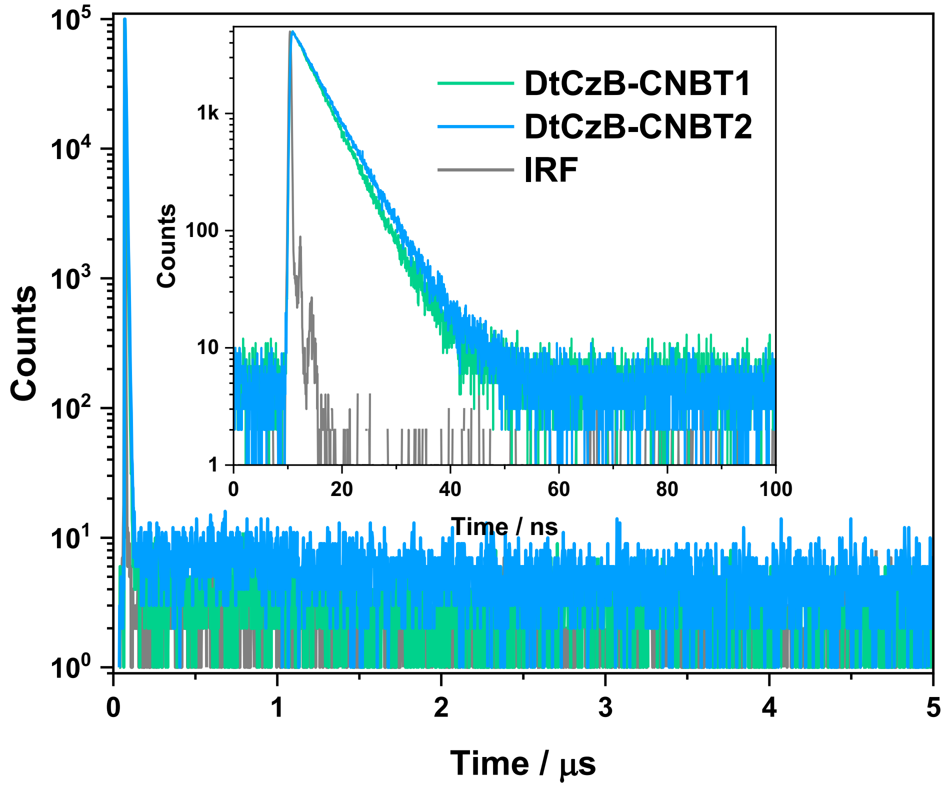

Figure **S27**. time-resolved PL decays for a) **DtCzBN-CNBT1**, and b) **DtCzBN-CNBT2** in dilute toluene (λ_{exc} = 379 nm).

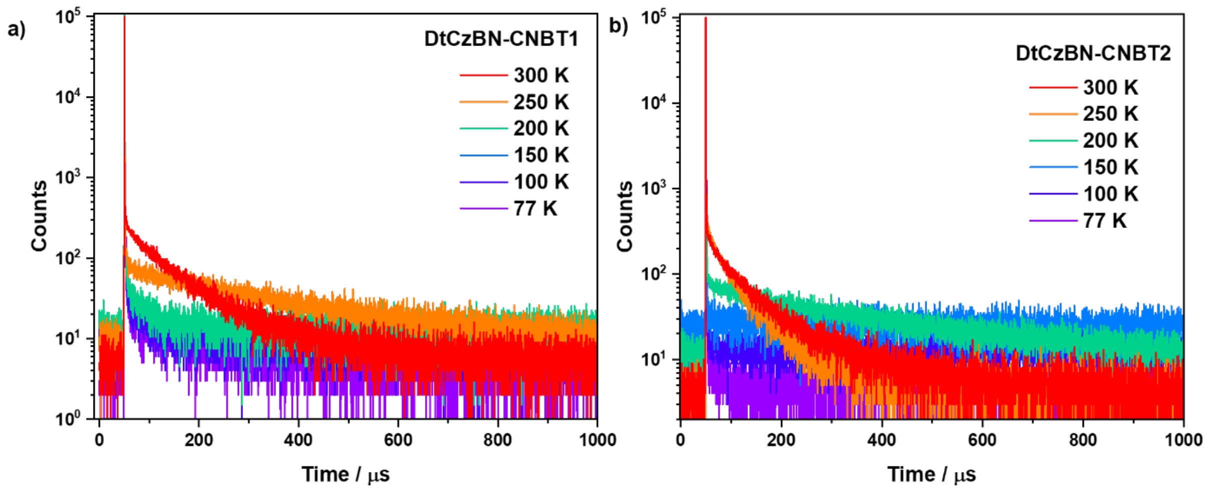

Figure **S28**. Temperature-dependent time-resolved PL decays for a) **DtCzBN-CNBT1**, and b) **DtCzBN-CNBT2** at 77 K in 15 wt% doped films in PhCzBCz (λ_{exc} = 379 nm).

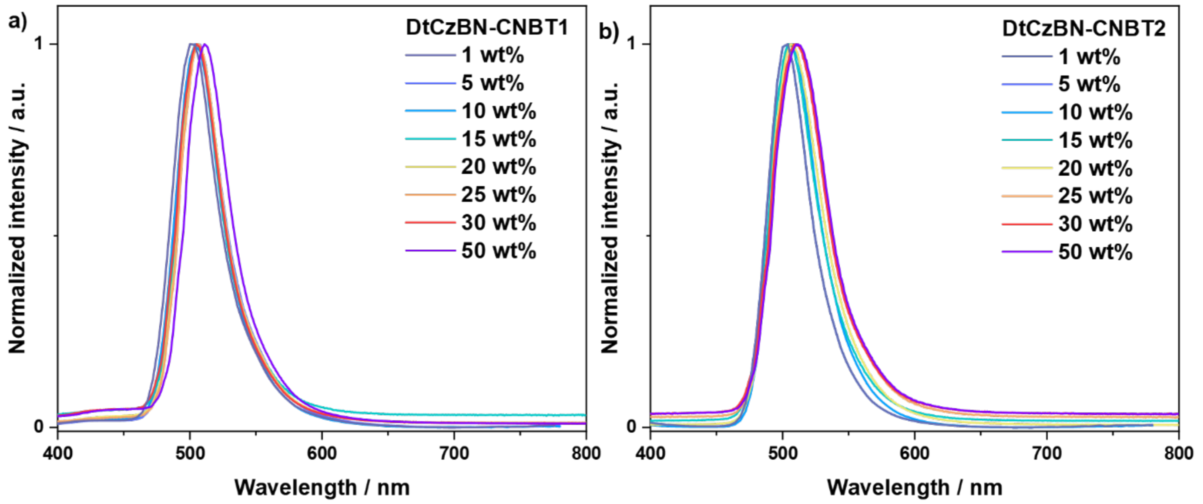

Figure **S29**. Concentration-dependent PL of a) **DtCzBN-CNBT1**, and b) **DtCzBN-CNBT2** in PhCzBCz doped films, λ_{exc} =340 nm.

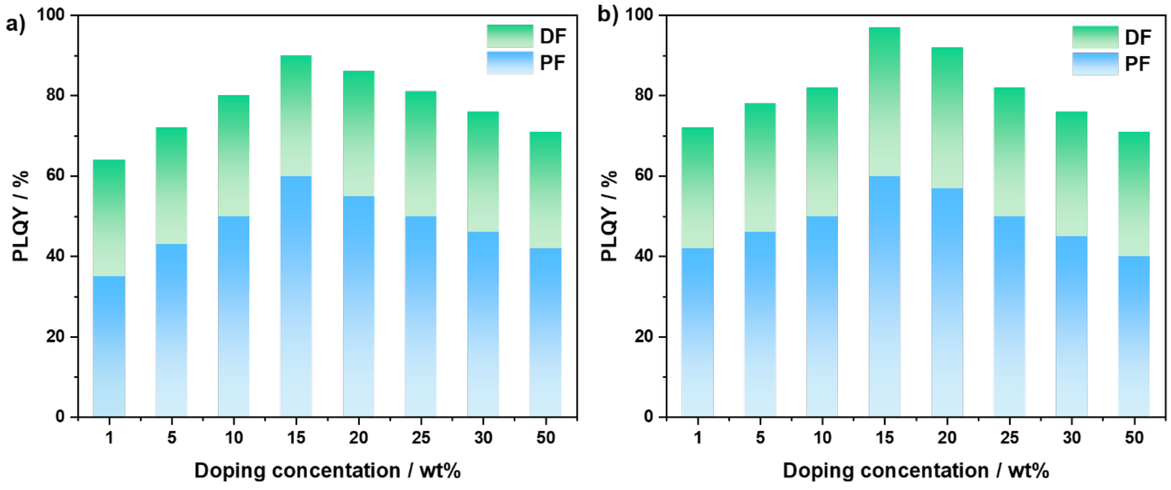

Figure **S30.** Concentration-dependent Φ_{PL} of a) **DtCzBN-CNBT1**, and b) **DtCzBN-CNBT2** in PhCzBCz doped films under nitrogen, λ_{exc} =340 nm.

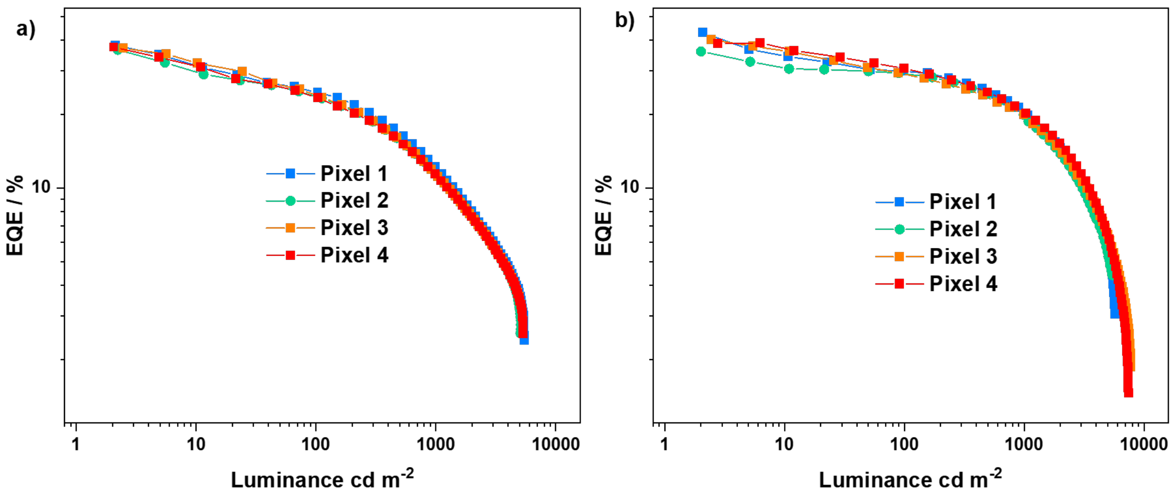

Figure **S31**. EQE versus luminance curves of four different devices with each of a) **DtCzBN-CNBT1**, and b) **DtCzBN-CNBT2** in 15 wt% doped PhCzBCz host as the EML.

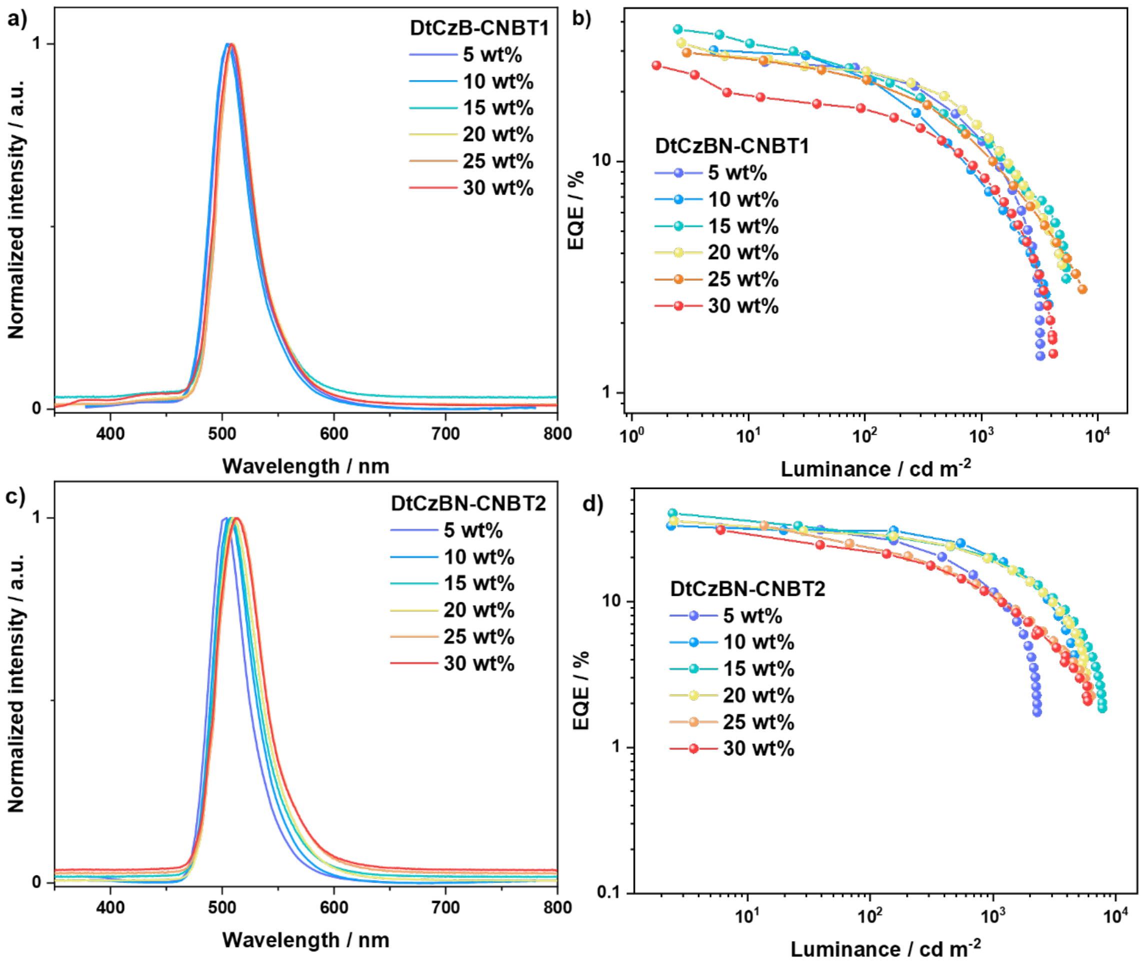

Figure **S32**. Eelectroluminescence spectra of a) **DtCzBN-CNBT1**, and c) **DtCzBN-CNBT2** and EQE versus luminance curves of b) **DtCzBN-CNBT1**, and d) **DtCzBN-CNBT2** in OLEDs with different doping concentration in PhCzBCz host.

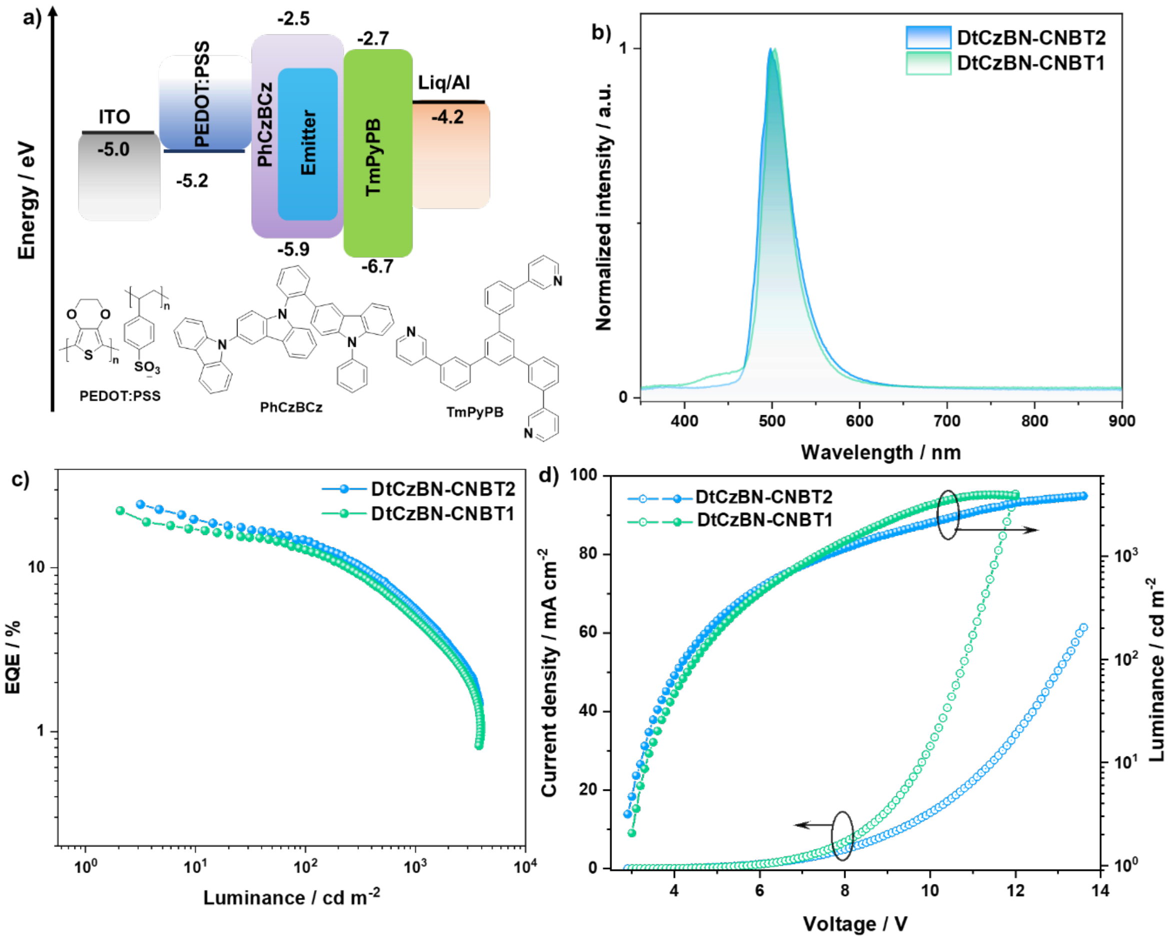

Figure **S33**. Optimized solution-processed devices based on **DtCzBN-CNBT1** and **DtCzBN-CNBT2**: a) Energy level diagram of the device structure and molecular structures of the materials used in the device b) EL spectra, c) EQE-luminance characteristics, d) Current density-voltage-luminance characteristics.

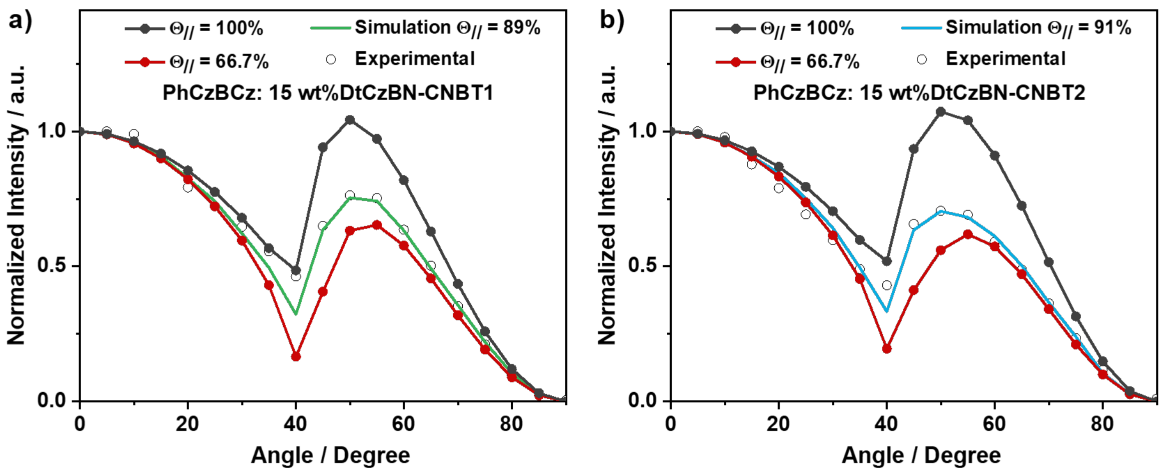

Figure **S34**. Angular-dependent *p*-polarized PL profiles of a) **DtCzBN-CNBT1** and b) **DtCzBN-CNBT2** in 15 wt% doped PhCzBCz film.

Table **S1**. Electroluminescent performance of reported green MR-TADF vacuum-deposited OLEDs

Emissive layer	V_{on} / V	λ_{EL} / nm	CIE	$EQE_{max}/EQE_{100}/EQE_{1000}$ / %	Lum_{max}/ cd m^{-2}	Lit.
15 wt% **DtCzBN-CNBT1** in PhCzBCz	3.1	508	(0.17, 0.66)	37.1/23.4/11.9	5 400	**This work**
15 wt% **DtCzBN-CNBT2** in PhCzBCz	3.1	508	(0.17, 0.66)	40.2/29.5/20.7	7 800	**This work**
8 wt% **TCzBN-S** in PhCzBCz	3.2	500	(0.13, 0.60)	30.0/28.6/16.9	4 000	ref[19]
8 wt% **TCzBN-SO** in PhCzBCz	2.7	512	(0.17, 0.69)	33.0/27.7/11.6	8 000	ref[19]
20 wt% **TCzBN-DPF** in SF3TRZ	3.1	501	(0.17, 0.60)	24.2/19.2/9.2	7 000	ref[20]
20 wt% **TCzBN-TMPh** in SF3TRZ	3.1	488	(0.10, 0.39)	18.6/6.9/0.8	1 100	ref[20]
20 wt% **TCzBN-oPh** in SF3TRZ	3.1	496	(0.11, 0.54)	24.2/14.7/3.7	3 000	ref[20]
2.5 wt% **DBTN-2** in SF3TRZ	2.8	520	(0.19, 0.74)	35.2/33.6/20.4	40 000	ref[21]
1 wt% **CzB2-M/P** in DOBNA-Tol	3.4	497	(0.12, 0.57)	26.7/17.5 /12.0	2 000	ref[22]
3 wt% **TW-BN** in mCBP	3.7	488	(0.14, 0.36)	27.8/25.4/10.7	9 500	ref[23]
3 wt% **TPh-BN** in mCBP	3.7	492	(0.10, 0.46)	28.9/25.1/15.6	13 000	ref[23]
3 wt% **pCz-BN** in mCBP	3.6	496	(0.13, 0.54)	27.2/25.6/12.2	11 000	ref[23]
3 wt% **mCz-BN** in mCBP	3.7	495	(0.15, 0.55)	25.9/24.1/14.0	13 000	ref[23]
3 wt % **Cz-PTZ-BN** in PhCzBCz	3.3	520	(0.26, 0.65)	27.6/26.2/17.3	29 000	ref[24]
3 wt % **2Cz-PTZ-BN** in PhCzBCz	3.3	516	(0.24, 0.63)	32.8/30.8/23.5	26 000	ref[24]
1 wt % **DBNO**:15 wt % 5TCzBN:PhCzBCz	3.0	504	(0.14, 0.53)	37.1/30.8/20.6	22 000	ref[25]

3 wt% **DtCzB-DPTRZ**:15 wt % 5TCzBN:PhCzBCz	2.8	528	(0.27, 0.60)	26.0/20.0/15.6	19 000	ref[26]
3 wt% **DtCzB-DPTRZ**:15 wt % 5TCzBN:PhCzBCz	2.8	508	(0.16, 0.65)	29.0/28.9/21.9	27 000	ref[26]
3 wt% **DtCzB-TPTRZ**:15 wt % 5TCzBN:PhCzBCz	2.8	540	(0.35, 0.63)	28.1/23.4/10.7	22 000	ref[26]
1 wt% **BN-DMAC** in mCBP	3.0	502	(0.14, 0.54)	21.1/19.7/12.5	4 000	ref[27]
1 wt% **BN-DPAC** in mCBP	3.5	504	(0.14, 0.56)	28.2/27.7/19.2	5 500	ref[27]
10 wt% **BN-CP1** in DMIC-TRZ	2.4	496	(0.09, 0.51)	38.0/31.9/16.1	52 000	ref[1]
10 wt% **BN-CP2**in DMIC-TRZ	2.4	499	(0.13, 0.59)	30.4/25.8/13.3	55 000	ref[1]
1 wt% **TCz-B** in mCBP	4.0	515	(0.21, 0.71)	29.2/24.7/9.4	3 000	ref[28]
1 wt% **(+)-BN4** in mCPCN	4.4	510	(0.19, 0.63)	20.6/20.5/10.7	3 300	ref[29]
1 wt% **(-)-BN4** in mCPCN	4.4	512	(0.21, 0.64)	19.0/16.6/10.1	3 000	ref[29]
3 wt% **(+)-BN5** in mCPCN	4.8	506	(0.17, 0.59)	22.0/15.3/10.9	3 000	ref[29]
3 wt% **(-)-BN5** in mCPCN	4.8	506	(0.17, 0.60)	26.5/17.6/11.1	3 000	ref[29]
1 wt% **2PXZBN** in mCBP/POT2T	4.8	522	(0.28, 0.64)	17.7/15.3/7.4	11 900	ref[30]
1 wt% **2PTZBN** in mCBP/POT2T	3.0	528	(0.28, 0.65)	25.5/21.7/17.2	16 700	ref[30]
4 wt% **SBSN** in mCBP	3.4	492	(0.10, 0.44)	17.6/17.6/12.0	22 800	ref[31]
4 wt% **DBON** in mCBP	3.6	510	(0.17, 0.68)	26.7/20.2/12.0	11 720	ref[31]
4 wt% **DBSN** in mCBP	3.7	556	(0.42, 0.57)	21.8/20.6/16.9	15 500	ref[31]
1 wt% **CzBSe** in mCBP	3.9	481	(0.10, 0.24)	23.9/23.4/20.0	5 000	ref[32]
5 wt% **Cz-BSeN** in mCBP	3.3	490	(0.13, 0.45)	20.3/16.9/12.7	11 200	ref[33]
5 wt% **DCz-BNN** in mCBP	3.8	500	(0.11, 0.44)	21.3/14.7/4.9	6 900	ref[33]
1 wt% **BN-Se** in DMIC-TRZ	2.4	506	(0.15, 0.62)	32.6/N.A./32.2	10^5	ref[34]
1 wt% **BNSSe** in DMIC-TRZ	2.4	515	(0.22, 0.66)	35.7/N.A./32.0	113 900	ref[35]
1 wt% **BNSeSe** in DMIC-TRZ	2.4	512	(0.19, 0.66)	36.8/N.A./34.0	108 200	ref[35]
5 wt% **BN-STO** in DMIC-TRZ	2.4	517	(0.19, 0.70)	40.1/39.0/28.1	93 300	ref[36]
5 wt% **BN-XTO** in DMIC-TRZ	2.5	516	(0.19, 0.69)	37.3/34.1/18.6	87 100	ref[36]
0.5 wt% **(SIPr)AuBN** in DMIC-TRZ	N.A.	511	(0.20, 0.69)	24.8/N.A./24.3	253 000	ref[37]
1 wt% **(IPr)AuBN** in DMIC-TRZ	N.A.	509	(0.16, 0.66)	24.0/N.A./20.2	117 000	ref[37]
1 wt % **(BzIPr)AuBN** in DMIC-TRZ	N.A.	510	(0.16, 0.68)	30.3/N.A./28.1	216 000	ref[37]
1 wt % **(PyIPr)AuBN** in DMIC-TRZ	N.A.	512	(0.18, 0.69)	27.6/N.A./20.5	151 000	ref[37]
1 wt % **(PzIPr)AuBN** in DMIC-TRZ	N.A.	515	(0.22, 0.67)	24.0/N.A./23.1	192 000	ref[37]
0.5 wt% **v-DABNA-CNMe** in DOBNA-Ph	3.2	504	(0.13, 0.65)	32.0/31.5/28.5	N.A.	ref[38]
3 wt% **DTB-BN2** in 2,6-DCzPPy	N.A.	497	(0.13, 0.49)	31.2/28.2/25.6	N.A.	ref[39]

4 wt% **VTCzBN** in 2,6-DCzPPy	3.9	499	(0.14, 0.56)	31.7/24.8/19.8	31 700	ref[40]
4 wt% **TCz-VTCzBN** in 2,6-DCzPPy	3.8	524	(0.22, 0.71)	32.2/18.0/16.0	38 700	ref[40]
0.5 wt% ***ω*-DABNA** in DOBNA-Ph	3.2	512	(0.13, 0.73)	31.1/30.8/29.4	N.A.	ref[41]
1 wt% **CzB4-oPh** in DOBNA-Ph	3.2	490	(0.09, 0.45)	28.7/27.8/25.8	N.A.	ref[42]
1 wt% **PTZBN1** :20 wt% USF in 2,6-DCzPPy	2.8	489	(0.14, 0.41)	32.7/25.9/16.5	30 800	ref[43]
1 wt% **(*P*)-Helicene-BN** in DMIC-TRZ	2.4	523	(0.26, 0.66)	31.5/29.6/18.7	71 500	ref[44]
1 wt% **(*M*)-Helicene-BN** in DMIC-TRZ	2.4	524	(0.26, 0.66)	30.7/28.3/17.9	77 200	ref[44]
20 wt% **IDAD-BNCz** in PhCzBCz	3.1	498	(0.10, 0.44)	34.3/31.8/14.0	6 000	ref[45]
20 wt% **DIDAD-BNCz** in PhCzBCz	3.0	490	(0.11, 0.45)	30.0/29.2/16.3	7 000	ref[45]
3 wt% **tCzBN-PQ** in 2,6-DCzPPy	3.8	524	(0.25, 0.67)	30.2/N.A./15.6	22 000	ref[46]
3 wt% **tCzBN-PQCz** in 2,6-DCzPPy	3.8	516	(0.20, 0.69)	35.1/N.A./22.6	27 000	ref[46]
3 wt% **TRZCzPh-BNCz** in CBP	3.2	513	(0.17, 0.68)	32.5/30.5/22.9	10 000	ref[47]
3 wt% **TRZTPh-BNCz** in CBP	3.2	513	(0.16, 0.70)	31.4/29.5/23.1	10 500	ref[47]

Table **S2**. Electroluminescent performance of reported solution-processed MR-TADF OLEDs

Emissive layer	V_{on} / V	λ_{EL} / nm	CIE	$EQE_{max}/EQE_{100}/EQE_{1000}$ / %	Lum_{max}/ cd m^{-2}	Lit.
15 wt% **DtCzBN-CNBT1** in PhCzBCz	3.5	508	(0.17, 0.66)	22.4/12.9/4.8	3 000	**This work**
15 wt% **DtCzBN-CNBT2** in PhCzBCz	3.4	507	(0.16, 0.67)	24.4/14.9/5.6	4 000	**This work**
2 wt% **tCzBN** in CzAcSF	4.0	490	(0.14, 0.34)	16.3/N.A./N.A.	2 500	ref[48]
1 wt% **tCzBN** : 30 wt% DMBN-PTC in 5TCzBN	3.1	490	(0.14, 0.34)	23.3/20.0/18.1	20 000	ref[49]
40 wt% **PCzBN3** in mCP	3.0	496	(0.12, 0.54)	17.5/9.1/2.5	2 800	ref[50]
0.5 wt% **v-DABNA** in PYD2:10 wt% Au-1	4.0	472	(0.14, 0.18)	16.6/N.A./14.4	8 000	ref[51]
1 wt% **V-DABNA-Mes** in TADF polymer C	3.2	480	(0.09, 0.21)	22.9/20.3/10.9	4 000	ref[52]
5 wt% **BON-D1** in mCP	3.5	488	(0.13, 0.44)	13.4/6.0/1.1	2 400	ref[53]
5 wt% **BON-D2** in mCP	3.6	487	(0.15,0.45)	14.9/10.1/4.0	4 300	ref[53]
2 wt% **OAB-ABP-1** in TADF polymer B	3.1	502	(0.12, 0.63)	21.8/19.6/17.4	10 000	ref[54]
2 wt% ***m*-Cz-BNCz** in D1-*t*Bu	4.2	514	(0.26, 0.61)	17.2/16.4/14.6	6 000	ref[55]
3 wt% **BN4** in mCPCN	4.4	510	(0.19, 0.63)	20.6/20.5/10.7	3 300	ref[29]
1 wt% **BN5** in mCPCN	4.9	506	(0.17, 0.59)	22.0/15.3/10.9	3 000	ref[29]

3 wt% **BN-36Cz-BN** in DMIC-TRZ	3.0	494	(0.11, 0.59)	22.6/N.A./6.2	7 000	ref[56]
3 wt% **BN-27Cz-BN** in DMIC-TRZ	3.0	498	(0.12, 0.55)	14.3/N.A./3.8	6 000	ref[56]
30 wt% **BSeN-Cz** in mCP	N.A.	486	(0.14, 0.40)	9.0/8.0/4.0	2 000	ref[57]
5 wt% **m-Cz-BNCz** in D1-tBu	4.2	521	(0.24, 0.68)	18.7/18.6/16.0	8 000	ref[55]
5 wt% **m-Cz-BNCz** in D1-OBu	3.2	519	(0.23, 0.68)	21.9/21.7/17.6	7 900	ref[55]
5 wt% **m-Cz-BNCz** in D2-OBu	2.8	519	(0.20, 0.67)	24.2/23.2/17.7	11 500	ref[55]
2 wt% **TCzBN-S** in PhCzBCz	3.1	500	(0.13, 0.59)	23.3/20.4/6.7	2 000	ref[19]
2 wt% **TCzBN-SO** in PhCzBCz	3.1	516	(0.19, 0.68)	25.5/19.3/6.9	3 000	ref[19]
5 wt% **QAO-Dad** in CBP	3.3	552	(0.41, 0.56)	19.3/N.A./11.5	10 000	ref[58]
16 wt% **4FlCzBN** in mCP	3.5	500	(0.11, 0.57)	10.9/6.7/4.6	2 800	ref[59]
2 wt% **3CzSF-BN** in PhCzBCz	2.6	522	(0.22, 0.72)	20.3/17.8/4.8	4 000	ref[60]
2 wt% **3CzSF-BN** : 30wt% 5TCzBN in PhCzBCz	2.6	519	(0.20, 0.71)	23.1/20.8/21.3	9 000	ref[60]
20 wt% **6TBN** in mCP	3.4	496	(0.09, 0.52)	23.0/16.5/6.4	7 900	ref[61]

References

[1] P. Jiang, J. Miao, X. Cao, H. Xia, K. Pan, T. Hua, X. Lv, Z. Huang, Y. Zou, C. Yang, *Adv. Mater.* **2022**, 34, 2106954.
[2] S. Grimme, *J. Comput. Chem.* **2004**, 25, 1463.
[3] a)S. Grimme, *Chem. Phys. Lett.* **1996**, 259, 128; b)S. Hirata, M. Head-Gordon, *Chem. Phys. Lett.* **1999**, 314, 291.
[4] X. Gao, S. Bai, D. Fazzi, T. Niehaus, M. Barbatti, W. Thiel, *J. Chem. Theory Comput.* **2017**, 13, 515.
[5] Gaussian 16, Revision C.01, M. J. Frisch, G. W. Trucks, H. B. Schlegel, G. E. Scuseria, M. A. Robb, J. R. Cheeseman, G. Scalmani, V. Barone, G. A. Petersson, H. Nakatsuji, X. Li, M. Caricato, A. V. Marenich, J. Bloino, B. G. Janesko, R. Gomperts, B. Mennucci, H. P. Hratchian, J. V. Ortiz, A. F. Izmaylov, J. L. Sonnenberg, Williams, F. Ding, F. Lipparini, F. Egidi, J. Goings, B. Peng, A. Petrone, T. Henderson, D. Ranasinghe, V. G. Zakrzewski, J. Gao, N. Rega, G. Zheng, W. Liang, M. Hada, M. Ehara, K. Toyota, R. Fukuda, J. Hasegawa, M. Ishida, T. Nakajima, Y. Honda, O. Kitao, H. Nakai, T. Vreven, K. Throssell, J. A. Montgomery Jr., J. E. Peralta, F. Ogliaro, M. J. Bearpark, J. J. Heyd, E. N. Brothers, K. N. Kudin, V. N. Staroverov, T. A. Keith, R. Kobayashi, J. Normand, K. Raghavachari, A. P. Rendell, J. C. Burant, S. S. Iyengar, J. Tomasi, M. Cossi, J. M. Millam, M. Klene, C. Adamo, R. Cammi, J. W. Ochterski, R. L. Martin, K. Morokuma, O. Farkas, J. B. Foresman, D. J. Fox, **2016**.
[6] M. Moral, L. Muccioli, W. J. Son, Y. Olivier, J. C. Sancho-García, *J. Chem. Theory Comput.* **2015**, 11, 168.
[7] V. GaussView, Semichem Inc., Shawnee Mission, KS 2019.
[8] a)C. Hättig, *J. Chem. Phys.* **2003**, 118, 7751; b)A. Hellweg, S. A. Grün, C. Hättig, *Phys. Chem. Chem. Phys.* **2008**, 10, 4119.
[9] O. S. Lee, M. Gather, E. Zysman-Colman, *Digit. Discov.* **2024**.
[10] N. M. O'Boyle, A. L. Tenderholt, K. M. Langner, *J. Comput. Chem.* **2008**, 29, 839.
[11] W. Humphrey, A. Dalke, K. Schulten, *J. Mol. Graph.* **1996**, 14, 33.
[12] C. S. D. J. Stone, University of Missouri-Rolla, 1998.
[13] J. D. Hunter, *Comput. Sci. Eng.* **2007**, 9, 90.
[14] N. M. O'Boyle, M. Banck, C. A. James, C. Morley, T. Vandermeersch, G. R. Hutchison, *J. Cheminform.* **2011**, 3, 33.
[15] N. M. O'Boyle, C. Morley, G. R. Hutchison, *Chem. Cent. J.* **2008**, 2, 5.
[16] N. G. Connelly, W. E. Geiger, *Chem. Rev.* **1996**, 96, 877.
[17] G. A. Crosby, J. N. Demas, *J. Phys. Chem.* **1971**, 75, 991.
[18] W. H. Melhuish, *J. Phys. Chem.* **1961**, 65, 229.
[19] F. Huang, Y.-C. Cheng, H. Wu, X. Xiong, J. Yu, X.-C. Fan, K. Wang, X.-H. Zhang, *Chem. Eng. J.* **2023**, 465, 142900.
[20] F. Huang, X.-C. Fan, Y.-C. Cheng, H. Wu, Y.-Z. Shi, J. Yu, K. Wang, C.-S. Lee, X.-H. Zhang, *Mater. Horiz.* **2022**, 9, 2226.
[21] X.-C. Fan, K. Wang, Y.-Z. Shi, Y.-C. Cheng, Y.-T. Lee, J. Yu, X.-K. Chen, C. Adachi, X.-H. Zhang, *Nat. Photon.* **2023**, 17, 280.
[22] S. Oda, W. Kumano, T. Hama, R. Kawasumi, K. Yoshiura, T. Hatakeyama, *Angew. Chem. Int. Ed.* **2021**, 60, 2882.
[23] F. Liu, Z. Cheng, L. Wan, Z. Feng, H. Liu, H. Jin, L. Gao, P. Lu, W. Yang, *Small* **2022**, 18, 2106462.
[24] F. Liu, Z. Cheng, Y. Jiang, L. Gao, H. Liu, H. Liu, Z. Feng, P. Lu, W. Yang, *Angew. Chem. Int. Ed.* **2022**, 61, e202116927.
[25] X. Cai, J. Xue, C. Li, B. Liang, A. Ying, Y. Tan, S. Gong, Y. Wang, *Angew. Chem. Int. Ed.* **2022**, 61, e202200337.
[26] Y. Xu, C. Li, Z. Li, J. Wang, J. Xue, Q. Wang, X. Cai, Y. Wang, *CCS Chem.* **2021**, 4, 2065.
[27] P. Jiang, L. Zhan, X. Cao, X. Lv, S. Gong, Z. Chen, C. Zhou, Z. Huang, F. Ni, Y. Zou, C. Yang, *Adv. Optical Mater.* **2021**, 9, 2100825.

[28] M. Yang, S. Shikita, H. Min, I. S. Park, H. Shibata, N. Amanokura, T. Yasuda, *Angew. Chem. Int. Ed.* **2021**, 60, 23142.
[29] X. Wu, J.-W. Huang, B.-K. Su, S. Wang, L. Yuan, W.-Q. Zheng, H. Zhang, Y.-X. Zheng, W. Zhu, P.-T. Chou, *Adv. Mater.* **2022**, 34, 2105080.
[30] T. Hua, L. Zhan, N. Li, Z. Huang, X. Cao, Z. Xiao, S. Gong, C. Zhou, C. Zhong, C. Yang, *Chem. Eng. J.* **2021**, 426, 131169.
[31] X.-F. Luo, H.-X. Ni, A.-Q. Lv, X.-K. Yao, H.-L. Ma, Y.-X. Zheng, *Adv. Optical Mater.* **2022**, 10, 2200504.
[32] I. S. Park, H. Min, T. Yasuda, *Angew. Chem. Int. Ed.* **2022**, 61, e202205684.
[33] Q. Li, Y. Wu, Q. Yang, S. Wang, S. Shao, L. Wang, *ACS Appl. Mater. Interfaces* **2022**, 14, 49995.
[34] X. Cao, K. Pan, J. Miao, X. Lv, Z. Huang, F. Ni, X. Yin, Y. Wei, C. Yang, *J. Am. Chem. Soc.* **2022**, 144, 22976.
[35] Y. X. Hu, J. Miao, T. Hua, Z. Huang, Y. Qi, Y. Zou, Y. Qiu, H. Xia, H. Liu, X. Cao, C. Yang, *Nat. Photon.* **2022**, 16, 803.
[36] Y. Hu, J. Miao, C. Zhong, Y. Zeng, S. Gong, X. Cao, X. Zhou, Y. Gu, C. Yang, *Angew. Chem. Int. Ed.* **2023**, 62, e202302478.
[37] S. Cai, G. S. M. Tong, L. Du, G. K.-M. So, F.-F. Hung, T.-L. Lam, G. Cheng, H. Xiao, X. Chang, Z.-X. Xu, C.-M. Che, *Angew. Chem. Int. Ed.* **2022**, 61, e202213392.
[38] S. Oda, T. Sugitani, H. Tanaka, K. Tabata, R. Kawasumi, T. Hatakeyama, *Adv. Mater.* **2022**, 34, 2201778.
[39] B. Lei, Z. Huang, S. Li, J. Liu, Z. Bin, J. You, *Angew. Chem. Int. Ed.* **2023**, 62, e202218405.
[40] X.-F. Luo, S.-Q. Song, H.-X. Ni, H. Ma, D. Yang, D. Ma, Y.-X. Zheng, J.-L. Zuo, *Angew. Chem. Int. Ed.* **2022**, 61, e202209984.
[41] S. Uemura, S. Oda, M. Hayakawa, R. Kawasumi, N. Ikeda, Y.-T. Lee, C.-Y. Chan, Y. Tsuchiya, C. Adachi, T. Hatakeyama, *J. Am. Chem. Soc.* **2023**, 145, 1505.
[42] Y. Sano, T. Shintani, M. Hayakawa, S. Oda, M. Kondo, T. Matsushita, T. Hatakeyama, *J. Am. Chem. Soc.* **2023**, 145, 11504.
[43] T. Hua, J. Miao, H. Xia, Z. Huang, X. Cao, N. Li, C. Yang, *Adv. Funct. Mater.* **2022**, 32, 2201032.
[44] W. Yang, N. Li, J. Miao, L. Zhan, S. Gong, Z. Huang, C. Yang, *CCS Chem.* **2022**, 4, 3463.
[45] J.-M. Jin, D. Liu, W.-C. Chen, C. Shi, G. Chen, X. Wang, L. Xing, W. Ying, S. Ji, Y. Huo, S.-J. Su, *Angew. Chem. Int. Ed.* **2024**, 63, e202401120.
[46] X. Song, S. Shen, S. Zou, Y. Wang, F. Guo, S. Gao, Y. Zhang, *Chem. Eng. J.* **2024**, 481, 148794.
[47] Y. Liu, X. Xiao, Z. Huang, D. Yang, D. Ma, J. Liu, B. Lei, Z. Bin, J. You, *Angew. Chem. Int. Ed.* **2022**, 61, e202210210.
[48] S. Xu, Q. Yang, Y. Zhang, H. Li, Q. Xue, G. Xie, M. Gu, J. Jin, L. Huang, R. Chen, *Chin. Chem. Lett.* **2021**, 32, 1372.
[49] D. Liu, Y. He, W. Qiu, X. Peng, M. Li, D. Li, J. Pu, J. Yang, Y. Gan, G. Yang, G. Sun, C. Shen, X. Cai, S.-J. Su, *Adv. Funct. Mater.* **2023**, 33, 2301327.
[50] T. Wang, Y. Zou, Z. Huang, N. Li, J. Miao, C. Yang, *Angew. Chem. Int. Ed.* **2022**, 61, e202211172.
[51] D. Zhou, S. Wu, G. Cheng, C.-M. Che, *J. Mater. Chem. C* **2022**, 10, 4590.
[52] S. Oda, B. Kawakami, Y. Yamasaki, R. Matsumoto, M. Yoshioka, D. Fukushima, S. Nakatsuka, T. Hatakeyama, *J. Am. Chem. Soc.* **2022**, 144, 106.
[53] J. Liu, L. Chen, X. Wang, Q. Yang, L. Zhao, C. Tong, S. Wang, S. Shao, L. Wang, *Macromol. Rapid Commun.* **2022**, 43, 2200079.
[54] N. Ikeda, S. Oda, R. Matsumoto, M. Yoshioka, D. Fukushima, K. Yoshiura, N. Yasuda, T. Hatakeyama, *Adv. Mater.* **2020**, 32, 2004072.
[55] R. Ma, Z. Ma, X. Wang, Z. Si, Q. Duan, S. Shao, *Chem. Eng. J.* **2022**, 447, 137517.
[56] T. Wang, X. Yin, X. Cao, C. Yang, *Angew. Chem. Int. Ed.* **2023**, 62, e202301988.
[57] L. Yang, P. Wang, K. Zhang, S. Wang, S. Shao, L. Wang, *Dyes Pigm.* **2023**, 216, 111371.

[58] Y. Yuan, X. Tang, X.-Y. Du, Y. Hu, Y.-J. Yu, Z.-Q. Jiang, L.-S. Liao, S.-T. Lee, *Adv. Optical Mater.* **2019**, 7, 1801536.
[59] N. Peethani, N. Y. Kwon, C. W. Koh, S. H. Park, J. M. Ha, M. J. Cho, H. Y. Woo, S. Park, D. H. Choi, *Adv. Optical Mater.* **2024**, 12, 2301217.
[60] X. Zhuang, B. Liang, C. Jiang, S. Wang, H. Bi, Y. Wang, *Adv. Optical Mater.* **2024**, *12*, 2400490.
[61] F.-M. Xie, H.-Z. Li, K. Zhang, H.-Y. Wang, Y.-Q. Li, J.-X. Tang, *ACS Appl. Mater. Interfaces* **2023**, 15, 39669.
